# A Lightweight Student Network with Dynamic Multi-Teacher Distillation for Optical Remote Sensing Object Detection

**DOI:** 10.3390/s26144599

**Published:** 2026-07-20

**Authors:** Jiarui Cai, Xudong Su, Haojun Deng, Jun Deng

**Affiliations:** School of Electronic Engineering, Xidian University, North Campus: No. 2 South Taibai Road, Xi’an 710071, China; 24022100026@stu.xidian.edu.cn (X.S.); denghaojun@stu.kust.edu.cn (H.D.); dengjun@xidian.edu.cn (J.D.)

**Keywords:** optical remote sensing object detection, lightweight object detection, YOLO11, geometrically decoupled regression tower, multi-teacher knowledge distillation, dynamic teacher weighting

## Abstract

Optical remote sensing object detection faces challenges such as large variations in scale, slender and direction-sensitive targets, complex backgrounds, and limited deployment resources. This paper proposes a lightweight geometrically decoupled student network with a dynamic multi-teacher distillation framework. Based on YOLO11n, the student detector keeps the original classification branch while redesigning the regression branches at different scales. Lightweight regression towers are used for the shallow and deep branches, whereas a geometrically decoupled regression tower is introduced only at the intermediate branch to enhance localization for slender and orientation-sensitive objects with limited extra cost. A geometry-adaptive box loss is further employed to stabilize localization training. For knowledge transfer, three specialized teachers are constructed for semantic classification, geometric regression, and structural topology supervision. A branch-decoupled adaptive weighting strategy dynamically integrates their complementary knowledge for classification and regression distillation. Experiments on DIOR show that the proposed model reduces parameters by 6.8% and GFLOPs by 13.9%, while improving mAP50 by 0.23 percentage points over YOLO11n. Validation on NWPU VHR-10 and deployment tests using PT, ONNX, and TensorRT further demonstrate improved accuracy–efficiency trade-offs and practical inference acceleration.

## 1. Introduction

Optical remote sensing object detection is a fundamental task in aerial and satellite image understanding, with important applications in airport monitoring, port scheduling, traffic management, disaster assessment, and land resource surveying. Compared with natural image object detection, optical remote sensing images typically exhibit large imaging scale spans, complex spatial target distributions, significant intra-class variations, and strong inter-class similarities [1,2]. Moreover, optical remote sensing objects often appear in dense configurations, with arbitrary orientations, slender shapes, and cluttered backgrounds, demanding not only stable semantic discrimination capability but also strong geometric localization and structural representation abilities [2,3]. Therefore, how to achieve a favorable trade-off among detection accuracy, localization robustness, and deployment efficiency in complex optical remote sensing scenarios remains a key research challenge in optical remote sensing image understanding.

In recent years, substantial efforts have been devoted to multi-scale modeling, geometric modeling, and complex-scenario adaptation for optical remote sensing object detection. On the methodological side, Clustered Object Detection [4] alleviates inefficiency caused by uniform tiling in large-scale optical remote sensing imagery via clustered region processing; Oriented R-CNN [5] improves arbitrary-oriented detection performance through high-quality rotated proposals; Oriented RepPoints [6] employs adaptive point sets to capture geometric structures of aerial targets; Dot Distance [7] addresses the sensitivity of small targets to localization offsets from a distance-metric perspective. These studies indicate that performance bottlenecks in optical remote sensing object detection stem not only from semantic recognition but also critically from object scale, shape structure, orientation response, and localization quality.

With the increasing deployment of UAVs, airborne platforms, and edge devices in optical remote sensing applications, detection models must achieve high accuracy while maintaining low parameter count, computational cost, and real inference latency. YOLO detectors, adopting a one-stage paradigm, offer a favorable accuracy–speed balance and are thus widely adopted in real-time vision and optical remote sensing detection tasks. YOLOv10 [8] further explores an end-to-end real-time detection framework to reduce post-processing dependence and improve deployment efficiency. YOLO11 [9], released by Ultralytics as a successor to previous YOLO series, introduces further optimizations in feature extraction, detection head design, and computational efficiency, where modules such as C3k2, SPPF, and C2PSA have been identified in recent works as key components for real-time detection. In addition, YOLO26n [10] is an official nano-level baseline from the same Ultralytics codebase for reference comparison. Consequently, constructing lightweight detectors for optical remote sensing based on the official Ultralytics framework [10] holds clear engineering feasibility and practical value.

Existing lightweight optical remote sensing detection methods typically reduce model complexity via lightweight backbones, attention enhancement, feature fusion, detection head compression, network pruning, or knowledge distillation, while striving to preserve detection performance [11,12,13]. Among these, knowledge distillation enables transferring representational capacity from a teacher model to a lightweight student model without increasing inference overhead, making it widely adopted for efficient detector training. In object detection, fine-grained feature imitation, instance-level distillation, and localization distillation have demonstrated that detection-specific distillation strategies effectively enhance student models’ classification and localization capabilities [14,15,16]. In optical remote sensing object detection, instance-aware distillation and feature distillation for airborne scenarios further confirm that distillation training helps mitigate performance degradation of lightweight models under small targets, complex backgrounds, and large instance variations [17,18].

However, directly applying existing lightweight and distillation strategies to optical remote sensing detection still faces limitations. First, the capabilities most vulnerable to model compression in optical remote sensing scenarios are not solely semantic classification representations but also geometric localization—especially for medium-scale, slender, and direction-sensitive targets. Uniform compression across all three-scale detection branches may weaken bounding box regression capacity at critical scales. Second, single-teacher or fixed-weight distillation often fails to simultaneously accommodate complementary knowledge such as semantic classification, geometric regression, and structural continuity, whereas the demand for teacher knowledge varies across categories, scales, and background conditions in optical remote sensing. Finally, existing lightweight methods often emphasize theoretical reductions in parameters or GFLOPs, while rarely validating actual inference gains across different deployment pipelines (e.g., PT, ONNX, and TensorRT).

**Figure 1:**
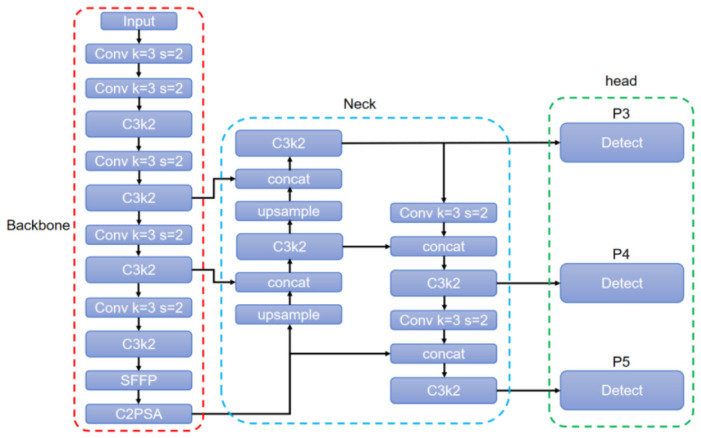
Structure of YOLO11.

To address the above issues, this paper investigates lightweight structural design and complementary knowledge transfer, proposing a lightweight geometrically decoupled student network with a multi-teacher adaptive distillation framework for optical remote sensing object detection. Following a two-stage principle of “structure-first, distillation-second”, we first differentiate the regression branches across scales on the YOLO11n backbone, as illustrated in Figure 1: lightweight regression structures are adopted in shallow (P3) and deep (P5) branches to reduce complexity, while a geometrically decoupled regression tower is introduced in the intermediate (P4) branch, where geometric cues are more salient, to enhance localization for medium-scale and slender targets.

Subsequently, to compensate for the knowledge degradation caused by lightweight compression, three heterogeneous teachers are constructed from complementary perspectives. Specifically, the semantic classification teacher is introduced to enhance category-discriminative knowledge and suppress background interference; the geometric regression teacher is designed to provide localization-oriented supervision for objects with large aspect-ratio variation and higher bounding-box regression difficulty; and the structural topology teacher is used to transfer contextual and relational knowledge for targets with weak appearance cues or complex surrounding structures. This three-teacher design is motivated by the fact that optical remote sensing detection involves heterogeneous difficulties, including small object scale, elongated object shape, and cluttered background interference, which are difficult to fully address using a single teacher model.

To further avoid redundant or conflicting supervision among heterogeneous teachers, a classification–regression separated multi-teacher adaptive distillation strategy is employed to dynamically balance their contributions during student training. The rationality of introducing these three teachers and the necessity of their dynamic integration are further examined in the attribute-oriented group analysis in Section 4.3.2, where small-target, elongated-target, and complex-background groups are constructed to verify whether different teachers provide complementary gains under different detection difficulties. The main contributions of this work are as follows:

1. We propose a lightweight student network tailored for optical remote sensing object detection. Built upon YOLO11n, the network differentiates regression branches across three detection heads: lightweight regression towers are employed in shallow (P3) and deep (P5) branches to reduce redundant computation, while a geometrically decoupled regression tower is constructed in the intermediate (P4) branch to better address common challenges in optical remote sensing, such as slender morphology, orientation sensitivity, and structural continuity.
2. We construct a three-teacher adaptive distillation framework comprising a semantic classification teacher, a geometric regression teacher, and a structural topology teacher, and design a classification–regression separated, multi-teacher adaptive distillation strategy. This enables the student model to adaptively absorb more reliable teacher knowledge per branch, thereby mitigating supervision bias inherent in single-teacher or fixed-weight distillation.
3. We conduct structural ablation, distillation mechanism analysis, and deployment efficiency evaluation on the DIOR dataset, supplemented by cross-dataset validation on NWPU VHR-10. Experimental results demonstrate that the proposed method further improves the trade-off among detection accuracy, model complexity, and practical inference efficiency over the compact YOLO11n baseline.

## 2. Related Work

### 2.1. Optical Remote Sensing Object Detection and Geometric Modeling

Optical remote sensing object detection is an important application of computer vision in the field of Earth observation, aiming to accurately localize target instances and recognize their categories in large-scale optical remote sensing images. Early studies typically relied on hand-crafted features and traditional classifiers to accomplish detection tasks, but these methods had limited adaptability to complex scene variations and changes in target morphology. With the development of deep learning-based object detection frameworks, optical remote sensing object detection has achieved significant progress in terms of public datasets and detection accuracy. For example, Cheng et al. [1] provided a systematic survey of object detection methods for optical remote sensing images; Li et al. [2] further constructed the DIOR dataset, providing an important benchmark for multi-category and multi-scale optical remote sensing object detection research; and the DOTA dataset has promoted the development of rotated object detection in large-scale aerial images [3]. Compared with natural image object detection, targets in optical remote sensing images usually exhibit more significant geometric variations and more complex spatial distributions.

Since the imaging perspective is mostly top-down or oblique, target orientations are no longer strongly constrained by the gravity direction and shooting angle as in natural images. For such targets, horizontal bounding box representations often fail to accurately fit target boundaries and tend to include redundant background information. This problem becomes more severe when targets are slender, densely arranged, or spatially adjacent, further increasing localization errors and category confusion. Therefore, detection methods for optical remote sensing objects have gradually begun to focus on orientation modeling, boundary representation, and geometric structure characterization. For example, Yang et al. [4] proposed a detection method for rotated targets to address clustered objects in aerial images; Xie et al. [5] proposed Oriented R-CNN, which further improves the localization capability for rotated object detection; and Oriented RepPoints proposed by Li et al. [6] models the orientation and shape characteristics of optical remote sensing targets from the perspective of point-set representation.

In summary, geometric modeling is an important means of improving the performance of optical remote sensing object detection. It not only improves the localization quality of direction-sensitive targets but also enhances the discriminative capability of models in scenarios involving complex backgrounds, small objects, and densely distributed targets. In this paper, the idea of geometric decoupling is further introduced into the lightweight student network, enabling the model to more fully exploit the orientation and structural information of targets while controlling the number of parameters and computational cost, thereby providing a more reliable feature basis for subsequent knowledge distillation and efficient deployment.

### 2.2. Lightweight Object Detection and YOLO Detectors

As optical remote sensing applications gradually expand from offline analysis to UAVs, airborne platforms, and edge devices, the computational efficiency and deployment performance of object detection models have attracted increasing attention. YOLO detectors adopt a one-stage detection paradigm and achieve a favorable trade-off among detection accuracy, inference speed, and deployment convenience, making them widely used in real-time object detection tasks. Figure 1 presents the detailed architecture of the YOLO11 model.

YOLO11 [9], as a subsequent model released by Ultralytics, has been further optimized in terms of feature extraction modules, detection head design, and computational efficiency, with related studies summarizing and analyzing components such as C3k2, SPPF, and C2PSA. YOLO26 [10] is included as an additional nano-level baseline from the same Ultralytics codebase. The YOLO11n, YOLO11s, and YOLO26n used in this paper are all built based on the official Ultralytics implementation [10].

In the field of optical remote sensing object detection, lightweight methods mainly reduce model complexity through structural designs such as lightweight backbones, channel compression, depthwise separable convolutions, and efficient detection heads. On this basis, some studies further combine attention mechanisms, feature fusion, or knowledge distillation to compensate for the possible degradation of feature representation capability during lightweight design. For example, lightweight optical remote sensing detection methods based on YOLOv5s introduce attention mechanisms to enhance feature representation of optical remote sensing targets while maintaining low model complexity [11]; the CTAM method based on YOLOv8n improves contextual modeling capability for optical remote sensing targets by integrating local convolutional features and global Transformer attention [12]; and lightweight detection methods for UAV scenarios further combine efficient feature fusion and knowledge distillation to meet the real-time detection requirements of airborne platforms [13]. These studies indicate that lightweight optical remote sensing detection is not merely about reducing the number of parameters and computational cost, but rather about achieving a comprehensive trade-off among model complexity, feature representation capability, and detection accuracy.

However, for already compact nano-level detectors, further reducing parameters and computational cost while maintaining detection performance is more challenging. Simply compressing the network structure uniformly may weaken the localization capability of critical detection branches, especially when optical remote sensing images contain numerous small targets, slender targets, and targets against complex backgrounds. Therefore, the structural design of lightweight optical remote sensing detectors should not only pursue an overall reduction in complexity but also consider the functional differences among detection branches at different scales, allocating limited computational resources to structural positions that are more sensitive to detection performance.

### 2.3. Knowledge Distillation and Efficient Optical Remote Sensing Object Detection

Knowledge distillation is an important technique for improving the performance of lightweight student models. Unlike image classification, object detection involves foreground–background imbalance, category discrimination, bounding box localization, and multi-scale target matching; therefore, detector distillation needs to be designed according to the characteristics of detection tasks. Fine-Grained Feature Imitation [14] improves the representation capability of student detectors for key regions by emphasizing fine-grained feature imitation around target areas. General Instance Distillation [15] further transfers detection knowledge from teacher detectors at the instance level, making the distillation process more aligned with the object detection task itself. Localization Distillation [16] focuses on transferring localization distributions and bounding box uncertainty, demonstrating that bounding box regression knowledge is also crucial for student detectors. Recent CrossKD [19] further shows that different branches inside the detection head contain differentiated knowledge, and cross-head distillation can improve the learning effectiveness of student models.

Recent studies have further explored feature-based and masked knowledge distillation for object detection. DCSF-KD [20] exploits both channel-wise and spatial feature knowledge, showing that different feature dimensions contain complementary information for detector compression. DMKD [21] improves feature-based detector distillation through dual masking augmentation, where spatially important regions and channel-wise informative cues are jointly considered. SAMKD [22] further introduces spatial-aware adaptive masking to enhance object-aware feature reconstruction. These methods demonstrate the effectiveness of transferring fine-grained feature knowledge from teacher detectors. However, they mainly focus on feature selection, feature reconstruction, or channel-spatial masking under a single-teacher distillation paradigm, while the adaptive integration of heterogeneous knowledge from multiple specialized teachers remains insufficiently explored.

In the field of optical remote sensing object detection, knowledge distillation has also been employed to improve the performance of efficient detectors. Instance-Aware Distillation [17] introduces instance-aware information into the distillation process to address characteristics of optical remote sensing images, such as large target scale variations, numerous small targets, and complex backgrounds, thereby improving the performance of efficient detectors in optical remote sensing scenarios. Studies on airborne optical remote sensing detection [18] also show that combining lightweight structures with feature distillation can alleviate the accuracy degradation caused by model compression without increasing inference-stage overhead. These studies demonstrate the practical value of distillation training for improving lightweight optical remote sensing detectors.

Nevertheless, existing methods insufficiently consider the differences among various types of knowledge, such as classification semantics, geometric regression, and structural priors. For optical remote sensing object detection, targets of different categories and scales may have noticeably different demands for teacher knowledge. For instance, category discrimination under complex backgrounds relies more on stable semantic supervision, whereas detecting slender and direction-sensitive targets depends more on geometric localization and structural continuity information. Therefore, how to adaptively integrate complementary knowledge from multiple teachers according to the different requirements of classification and regression branches is a problem worth investigating for improving the performance of lightweight optical remote sensing detectors. Different from existing feature-based or masked distillation methods, our dynamic multi-teacher adaptive distillation framework explicitly decomposes teacher supervision into semantic, geometric, and structural knowledge, and adaptively assigns complementary supervision signals to the student detector without increasing inference-stage computational overhead.

## 3. Method Overview

### 3.1. Overall Framework

**Figure 2:**
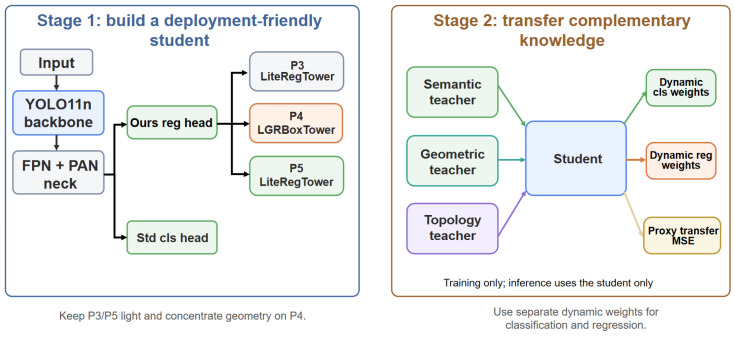
Overall framework of the lightweight student and dynamic multi-teacher distillation.

This paper proposes a lightweight geometrically decoupled student network with a multi-teacher adaptive distillation framework for optical remote sensing object detection. The overall method follows a two-stage principle of “structure-first, distillation-second”, as shown in Figure 2. In the first stage, a deployment-friendly lightweight student model is constructed based on YOLO11n. By differentially designing the regression branches in the three-scale detection heads, lightweight regression towers are adopted in the shallow (P3) and deep (P5) branches to reduce redundant computation, while a geometrically decoupled regression tower is introduced into the intermediate (P4) branch to model local neighborhoods, orientation responses, and topological structure information. This design reduces the number of parameters and computational cost while preserving geometric modeling capability at critical scales. In the second stage, a semantic classification teacher, a geometric regression teacher, and a structural topology teacher are introduced. A classification–regression separated multi-teacher adaptive distillation strategy is adopted to compute dynamic weights for the three teachers according to the different supervision requirements of the classification and regression branches, thereby enabling more fine-grained complementary knowledge transfer. The complementary knowledge from different teachers is transferred to the student model.

### 3.2. Lightweight Geometrically Decoupled Student Network

The student model does not apply a uniform lightweight design to the entire detection head; instead, it explicitly considers the functional heterogeneity of different detection scales. In YOLO-style multi-scale detection, the shallow (P3) branch mainly handles small objects. Although it preserves fine spatial resolution, the semantic representation and geometric context available at this level are relatively limited. Introducing a complex geometrically enhanced regression tower on (P3) would therefore bring considerable computational overhead, while the corresponding geometric gain may be limited due to insufficient high-level context. In contrast, the deep (P5) branch is responsible for large objects and contains stronger semantic information with a larger receptive field. The localization of large objects is generally more stable at this scale, and further strengthening the geometric regression tower on (P5) may lead to redundant computation rather than a clear accuracy improvement.

Based on this observation, we introduce the Lightweight Geometrically Decoupled Regression Box Tower (LGRBoxTower) only on the intermediate (P4) detection scale. The (P4) branch provides a more balanced trade-off between spatial resolution and semantic abstraction, making it more suitable for geometry-aware localization. In remote sensing object detection, many objects appear at medium scales or with complex aspect ratios and orientations, and their accurate localization requires both sufficient semantic discrimination and reliable geometric modeling. Therefore, enhancing the regression capability on (P4) can effectively improve localization quality while avoiding the excessive cost of applying the same geometric module to all detection scales.

**Figure 3:**
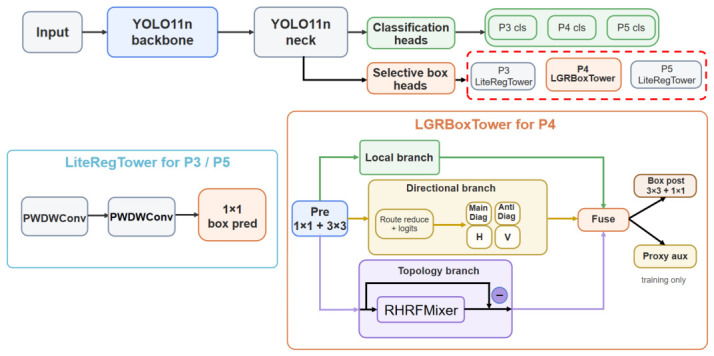
Structure of the student detector.

Specifically, the shallow (P3) and deep (P5) regression branches employ the Lightweight Regression Tower (LiteRegTower) to maintain computational efficiency, whereas the intermediate (P4) branch adopts LGRBoxTower to preserve stronger geometric modeling capability. Meanwhile, the original classification head of YOLO11 is kept unchanged. This selective design confines the additional complexity to the geometrically sensitive critical-scale branch and compensates for it through lightweight regression towers at both ends, thereby achieving a better balance between detection accuracy and computational efficiency. Figure 3 illustrates the architecture of the student network.

#### 3.2.1. Lightweight Regression Tower

LiteRegTower consists of two depthwise separable convolution blocks, i.e., PWDWConv(.), and one 1 × 1 prediction layer, where PWDWConv(.) is composed of depthwise convolution DWConv [23] and pointwise convolution PWConv [24]. Compared with a standard regression tower stacked with regular convolutions, this design can significantly reduce the number of parameters and GFLOPs while preserving basic localization capability. Let the input feature be x and the output bounding box distribution prediction be box. The computation process can be written.

(1) $$\mathrm{PWDWConv}\left(x\right)={\mathrm{PWConv}}_{1\times 1}\left({\mathrm{DWConv}}_{3\times 3}\left(x\right)\right)$$

(2) $$\mathrm{box}={\mathrm{Conv}}_{1\times 1}\left(\mathrm{PWDWConv}(\mathrm{PWDWConv}(x))\right)$$

#### 3.2.2. Geometrically Decoupled Regression

LGRBoxTower is the core component of the geometrically decoupled regression. Its detailed architecture is shown in Figure 3. The initial preprocessing stage contains only a 1×1 convolution and a 3×3 convolution. Subsequently, the processed feature is decomposed into three complementary paths. The local branch preserves neighborhood details through depthwise convolution and pointwise fusion; the orientation branch first generates low-dimensional routing features through route_reduce in Equation (11), then predicts four routing weights via route_logits, and adaptively combines the horizontal strip convolution, vertical strip convolution, main-diagonal band convolution, and anti-diagonal band convolution; the topology branch invokes RHRFMixer to capture structured channel interactions. Finally, the three branches are fused to obtain the geometry-enhanced feature feat. In the main branch, feat is mapped into a discrete bounding box distribution through the post layer. During training, feat is additionally equipped with a lightweight proxy auxiliary head, including a center proxy head and a boundary proxy head, which predict the center response map and boundary response map through 1×1 convolutions, respectively, providing additional spatial structure supervision for the geometrically fused feature.

Let the feature input to LGRBoxTower be denoted as x. The preprocessing, orientation routing, and multi-branch fusion of LGRBoxTower can be written as follows:

(3) $$x_{0}=\mathrm{Pre}\left(x\right)={\mathrm{Conv}}_{3\times 3}\left({\mathrm{Conv}}_{1\times 1}\left(x\right)\right)$$

(4) $$\begin{array}{cc} \mathrm{local}\_\mathrm{feat} & =\mathrm{localBranch}(x_{0});\\ \mathrm{dir}\_\mathrm{feat} & =\mathrm{DirectionBranch}(x_{0});\\ \mathrm{topo}\_\mathrm{feat} & =\mathrm{RHRFMixer}\left(x_{0}\right)-x_{0} \end{array}$$

(5) $$\begin{array}{cc} \mathrm{feat} & =\mathrm{Fuse}(\mathrm{local}\_\mathrm{feat},\mathrm{dir}\_\mathrm{feat},\mathrm{topo}\_\mathrm{feat})\\ \; & ={\mathrm{Conv}}_{3\times 3}\left({\mathrm{Conv}}_{1\times 1}\left(\mathrm{Concat}(\mathrm{local}\_\mathrm{feat},\mathrm{dir}\_\mathrm{feat},\mathrm{topo}\_\mathrm{feat})\right)\right) \end{array}$$

(6) $$\mathrm{box}={\mathrm{Conv}}_{1\times 1}\left({\mathrm{Conv}}_{3\times 3}\left(\mathrm{feat}\right)\right)$$

(7) $$Z_{\mathrm{center}}={\mathrm{Conv}}_{1\times 1}\left(\mathrm{feat}\right);\;Z_{\mathrm{boundary}}={\mathrm{Conv}}_{1\times 1}\left(\mathrm{feat}\right)$$

(8) $$\mathrm{Aux}\left(\mathrm{feat}\right)=\left\{\begin{array}{cc} \{Z_{\mathrm{center}},Z_{\mathrm{boundary}}\}, & \mathrm{training},\\ ⌀, & \mathrm{inference}. \end{array}\right.$$

where $x_{0}$ denotes the preprocessed feature, while local_feat, dir_feat, and topo_feat denote the features extracted by the local branch, orientation branch, and topology branch, respectively; box is the final bounding box regression output; $Z_{center}$ and $Z_{boundary}$ denote the center proxy prediction map and boundary proxy prediction map generated by the proxy auxiliary head during training, respectively. The subtraction operation extracts the topology-induced residual component, allowing the topology branch to focus on structural variations rather than duplicating the original feature response.

Furthermore, the functions localBranch(.) and DirectionBranch(.) are computed as follows:

(9) $$\mathrm{localBranch}\left(x_{0}\right)={\mathrm{Conv}}_{1\times 1}\left(\mathrm{SiLU}\left(\mathrm{BN}\left({\mathrm{DWConv}}_{3\times 3}\left(x_{0}\right)\right)\right)\right)$$

(10) $$\mathrm{DirectionBranch}\left(x_{0}\right)=\sum_{n=1}^{4}w_{n}⊙y_{n}$$

(11) $$\begin{array}{cc} w_{1},w_{2},w_{3},w_{4} & =\mathrm{route}\_\mathrm{logits}\left(\mathrm{route}\_\mathrm{reduce}(x_{0})\right)\\ \; & =\mathrm{Softmax}\left({\mathrm{Conv}}_{1\times 1}\left(\mathrm{SiLU}\left(\mathrm{BN}\left({\mathrm{Conv}}_{1\times 1}\left(x_{0}\right)\right)\right)\right)\right) \end{array}$$

(12) $$\begin{array}{cc} y_{1} & =y_{h}={\mathrm{Conv}}_{1\times 1}\left(\mathrm{SiLU}(\mathrm{BN}\left({\mathrm{DWConv}}_{1\times k}\left(x_{0}\right)\right))\right);\\ y_{2} & =y_{v}={\mathrm{Conv}}_{1\times 1}\left(\mathrm{SiLU}(\mathrm{BN}\left({\mathrm{DWConv}}_{k\times 1}\left(x_{0}\right)\right))\right);\\ y_{3} & =y_{d1}=\mathrm{SiLU}\left(\mathrm{BN}({\mathrm{MainDiagDWConv}}_{7\times 7}\left(x_{0}\right))\right);\\ y_{4} & =y_{d2}=\mathrm{SiLU}\left(\mathrm{BN}({\mathrm{AntiDiagDWConv}}_{7\times 7}\left(x_{0}\right))\right) \end{array}$$

where $w_{n}$ denotes the routing weight of the *n*-th directional branch, SiLU(.) [25], BN(.) [26], MainDiagDWConv(.) [27], and AntiDiagDWConv(.) [27] denote the activation function, normalization function, main-diagonal depthwise convolution, and anti-diagonal depthwise convolution, respectively; $y_{h}$, $y_{v}$, $y_{d1}$, and $y_{d2}$ denote the horizontal feature, vertical feature, main-diagonal feature, and anti-diagonal feature, respectively; ⊙ denotes element-wise multiplication.

**Figure 4:**
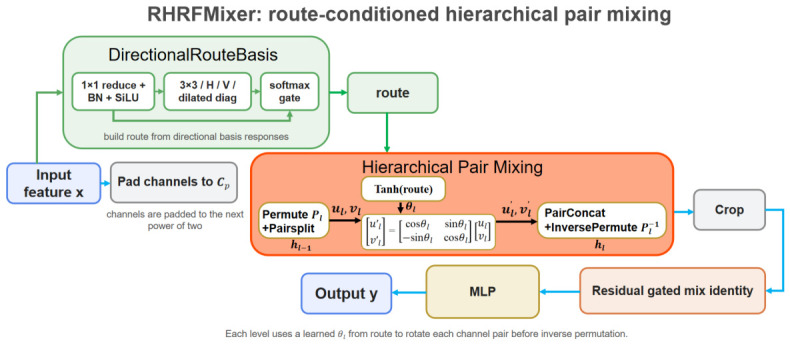
Structure of RHRFMixer with route-conditioned hierarchical pair mixing.

Furthermore, RHRFMixer($x_{0}$) in the topology branch is not a conventional channel reshuffling operation. Figure 4 illustrates the channel mixing mechanism of this process, whose core can be written as follows:

We decompose the RHRFMixer operator into four parts, as follows:

1. The number of channels is padded to $C_{p}=2^{\left\lceil \log_{2}C\right\rceil }$. If the number of channels satisfies $C\neq C_{p}$, zero-padding with Pad is performed along the channel dimension:
  (13) X=Pad(x)
  where x denotes the input feature, and X denotes the feature after padding.
2. Let the input feature be x. DirectionalRouteBasis is used to generate the routing feature:
  (14) $$z=\mathrm{SiLU}\left(\mathrm{BN}({\mathrm{Conv}}_{1\times 1}\left(x\right))\right)$$
  (15) $$\begin{array}{cc} w_{1},w_{2},w_{3},w_{4} & =W_{r}=\mathrm{Softmax}\left({\mathrm{Conv}}_{1\times 1}\left(\mathrm{SiLU}(\mathrm{BN}\left({\mathrm{DWConv}}_{3\times 3}\left(z\right)\right))\right)\right) \end{array}$$
  (16) $$\begin{array}{cc} r_{1} & ={\mathrm{DWConv}}_{3\times 3}\left(z\right);\\ r_{2} & ={\mathrm{DWConv}}_{1\times 5}\left(z\right);\\ r_{3} & ={\mathrm{DWConv}}_{5\times 1}\left(z\right);\\ r_{4} & ={\mathrm{DilatedDWConv}}_{3\times 3}\left(z,\;\mathrm{rate}=2\right) \end{array}$$
  (17) $$\mathrm{route}=\mathrm{DirectionalRouteBasis}\left(x\right)=\mathrm{SiLU}\left(\mathrm{BN}\left(\sum_{n=1}^{4}w_{n}⊙r_{n}\right)\right)$$
  where z denotes the preprocessed feature value; DilatedDWConv(.) [28] denotes dilated depthwise convolution.
3. The initial level feature is set as $h_{0}=X$. For the *l*-th level, we use $h_{l}$ to denote the input feature of the *l*-th level, where $h_{l}$ refers to the channel-padded feature X. A fixed bit-pair permutation $P_{l}$ is adopted to reorder the channels, where $P_{l}$ denotes the channel permutation rule of the *l*-th level:
  (18) $${\bar{h}}_{l}=\mathrm{Permute}\left(h_{l},P_{l}\right)=h_{l}\left[:,\;P_{l},:,:\right]$$
  (19) $$u_{l},v_{l}=\mathrm{PairSplit}\left({\bar{h}}_{l}\right)$$
  where PairSplit(·) denotes splitting ${\bar{h}}_{l}$ into adjacent channel pairs along the channel dimension:
  (20) $${\bar{h}}_{l}\in R^{B\times C_{p}\times H\times W},\;{\tilde{h}}_{l}=\mathrm{reshape}\left({\bar{h}}_{l}\right)\in R^{B\times \frac{C_{p}}{2}\times 2\times H\times W}$$
  (21) $$u_{l}={\tilde{h}}_{l}\left[:,:,0,:,:\right],\;v_{l}={\tilde{h}}_{l}\left[:,:,1,:,:\right]$$
  Furthermore, after channel pairing, rotational mixing is performed according to $\theta_{l}$ predicted from the routing feature:
  (22) $$\theta_{l}=\frac{\pi }{4}\tanh\left({\mathrm{Conv}}_{1\times 1}\left(\mathrm{route}\right)\right)$$
  (23) $$\left[\begin{array}{c} u_{l}^{\prime }\\ v_{l}^{\prime } \end{array}\right]=\left[\begin{array}{cc} \cos\theta_{l} & \sin\theta_{l}\\ -\sin\theta_{l} & \cos\theta_{l} \end{array}\right]\left[\begin{array}{c} u_{l}\\ v_{l} \end{array}\right]$$
  (24) $$h_{l+1}=\mathrm{InversePermute}\left(\mathrm{PairConcat}\left(u_{l}^{\prime },v_{l}^{\prime }\right),P_{l}^{-1}\right)$$
  (25) $$\mathrm{InversePermute}\left(z,P_{l}^{-1}\right)=z\left[:,\;P_{l}^{-1},:,:\right]$$
  (26) $$\mathrm{PairConcat}\left(u_{l}^{\prime },v_{l}^{\prime }\right)=\mathrm{reshape}\left(\mathrm{stack}\left(\left[u_{l}^{\prime },v_{l}^{\prime }\right],\dim=2\right),\left(B,C_{p},H,W\right)\right)$$
  where route denotes the routing feature generated by DirectionalRouteBasis; Permute(.) denotes the channel reordering function; $u_{l}$ and $v_{l}$ denote the two channel features split by the PairSplit function, respectively; $\theta_{l}$ denotes the rotation angle predicted from the routing feature; InversePermute(.) denotes inverse channel reordering; PairConcat(.) denotes channel concatenation.
4. Further enhancement is performed through a low-rank MLP:
  (27) $$\bar{x}=\mathrm{Crop}\left(h_{L}\right)$$
  (28) $$y_{\mathrm{mix}}=x+\sigma \left(\beta_{m}\right)\left(\bar{x}-x\right)$$
  (29) $$y_{1}=\mathrm{MLP}\left(y_{\mathrm{mix}}\right)={\mathrm{Conv}}_{1\times 1}\left(\mathrm{SiLU}({\mathrm{Conv}}_{1\times 1}\left(y_{\mathrm{mix}}\right))\right)$$
  (30) $$y=y_{\mathrm{mix}}+\sigma \left(\beta_{f}\right)y_{1}$$
  where $h_{L}$ denotes the feature after all levels; Crop(.) denotes channel cropping; σ(·) denotes the Sigmoid activation function; MLP(.) denotes the low-rank feed-forward enhancement module; $\beta_{m}$ and $\beta_{f}$ denote learnable scalar parameters.

#### 3.2.3. Geometric Adaptive Box Loss

To stabilize localization training for elongated and small targets under the lightweight regression design, this paper introduces a geometric adaptive box loss as an auxiliary bounding-box regression constraint. This auxiliary loss explicitly introduces center-offset and shape-offset constraints into the regression objective, and constructs a geometry-aware weighting term according to the aspect ratio and spatial size of each target. Different from the original CIoU-based box loss used in the baseline implementation, the proposed geometric adaptive box loss adopts IoU as the basic overlap term and assigns larger regression weights to targets with large aspect ratios or small spatial extents, thereby strengthening the learning of targets with strong geometric attributes.

The computation process of the geometric adaptive box loss is as follows. For the *j*-th foreground sample, the predicted bounding box and the assigned ground-truth bounding box are represented in the following format:

(31) $${\hat{b}}_{j}=\left({\hat{x}}_{1j},{\hat{y}}_{1j},{\hat{x}}_{2j},{\hat{y}}_{2j}\right)$$

(32) $$b_{j}^{*}=\left(x_{1j}^{*},y_{1j}^{*},x_{2j}^{*},y_{2j}^{*}\right)$$

where ${\hat{b}}_{j}$ and $b_{j}^{*}$ denote the predicted box and the assigned ground-truth box of the *j*-th foreground sample, respectively. Based on the xyxy box representation, the center coordinates and width-height sizes of the predicted and target boxes are computed as follows:

(33) $${\hat{c}}_{j}=\left({\hat{c}}_{xj},{\hat{c}}_{yj}\right)=\left(\frac{{\hat{x}}_{1j}+{\hat{x}}_{2j}}{2},\frac{{\hat{y}}_{1j}+{\hat{y}}_{2j}}{2}\right)$$

(34) $$c_{j}^{*}=\left(c_{xj}^{*},c_{yj}^{*}\right)=\left(\frac{x_{1j}^{*}+x_{2j}^{*}}{2},\frac{y_{1j}^{*}+y_{2j}^{*}}{2}\right)$$

(35) $${\hat{s}}_{j}=\left({\hat{w}}_{j},{\hat{h}}_{j}\right)=\left({\hat{x}}_{2j}-{\hat{x}}_{1j},{\hat{y}}_{2j}-{\hat{y}}_{1j}\right)$$

(36) $$s_{j}^{*}=\left(w_{j}^{*},h_{j}^{*}\right)=\left(x_{2j}^{*}-x_{1j}^{*},y_{2j}^{*}-y_{1j}^{*}\right)$$

The IoU between the predicted box and the assigned ground-truth box is defined as follows:

(37) $$u_{j}=\mathrm{IoU}\left({\hat{b}}_{j},b_{j}^{*}\right)$$

The center-offset loss normalizes the absolute center displacement by the target width and height, which can be written as follows:

(38) $$L_{\mathrm{ctr}}^{j}=\frac{|{\hat{c}}_{xj}-c_{xj}^{*}|}{w_{j}^{*}+\varepsilon }+\frac{|{\hat{c}}_{yj}-c_{yj}^{*}|}{h_{j}^{*}+\varepsilon }$$

The shape-offset loss measures the logarithmic deviation between the predicted and target width-height sizes:

(39) $$L_{\mathrm{shape}}^{j}=\left|\log\frac{{\hat{w}}_{j}+\varepsilon }{w_{j}^{*}+\varepsilon }\right|+\left|\log\frac{{\hat{h}}_{j}+\varepsilon }{h_{j}^{*}+\varepsilon }\right|$$

To emphasize targets with strong geometric attributes, a geometric adaptive weight is constructed according to the aspect ratio and spatial size of the assigned target box:

(40) $$g_{j}=\mathrm{clip}\left(1+a\left|\log\frac{w_{j}^{*}+\varepsilon }{h_{j}^{*}+\varepsilon }\right|+\frac{b}{\sqrt{w_{j}^{*}h_{j}^{*}+\varepsilon }},1,g_{\max}\right)$$

In addition, the foreground regression weight of the *j*-th sample is obtained from the target scores assigned by the task-aligned assigner:

(41) $$q_{j}=\sum_{c=1}^{N_{c}}t_{j,c}$$

where $t_{j,c}$ denotes the assigned target score of the *j*-th foreground sample for the *c*-th category, and $N_{c}$ denotes the number of object categories. The final geometric adaptive box regression loss is defined as follows:

(42) $$L_{\mathrm{box}}^{\mathrm{geo}}=\frac{1}{S}\sum_{j\in \Omega }q_{j}g_{j}\left[1-u_{j}+\lambda_{c}L_{\mathrm{ctr}}^{j}+\lambda_{s}L_{\mathrm{shape}}^{j}\right]$$

where Ω denotes the foreground sample set; $S=\max(\sum_{j\in \Omega }\sum_{c=1}^{N_{c}}t_{j,c},1)$ is the normalization factor corresponding to the sum of assigned target scores; $q_{j}$ is the foreground regression weight of the *j*-th sample; $g_{j}$ is the geometric adaptive weight; $u_{j}$ denotes the IoU between the predicted box and the assigned ground-truth box; $L_{\mathrm{ctr}}^{j}$ and $L_{\mathrm{shape}}^{j}$ denote the center-offset loss and shape-offset loss, respectively; $\lambda_{c}$ and $\lambda_{s}$ are the weighting coefficients of the center-offset and shape-offset terms; *a* controls the contribution of the aspect-ratio term; *b* controls the contribution of the small-object term; $g_{\max}$ is the upper bound of the geometric adaptive weight; and ε is a small constant used for numerical stability. In all experiments, the hyperparameters are set to $\lambda_{c}=0.25$, $\lambda_{s}=0.10$, a=0.30, b=0.20, $g_{\max}=2.0$, and $\varepsilon =10^{-6}$. The width and height terms $w_{j}^{*}$ and $h_{j}^{*}$ are computed in the same stride-normalized coordinate space as the decoded boxes used by the detection loss.

### 3.3. Construction Logic of the Three Specialized Teachers

The distillation stage is not a simple “teacher replacement”. Instead, the loss implementation explicitly loads a semantic classification teacher, a geometric regression teacher, and a structural topology teacher, and computes teacher weights separately for the classification and regression branches. Therefore, the teacher system is not an externally attached experimental configuration, but an intrinsic component of the proposed method.

It should be noted that the three teachers are not obtained by online co-training with the student. Each teacher is trained independently on the DIOR training set before the distillation stage. For all teacher models, the same training protocol as the student model is adopted, including the input image size, optimizer, learning rate, training epochs, batch size, data augmentation strategy, and random seed settings. Specifically, each teacher is trained with the same three random seeds used in the student experiments, and the checkpoint with the best validation mAP50 is selected as the final teacher checkpoint. During student distillation, all teacher networks are loaded in evaluation mode and their parameters are frozen. Therefore, gradients are propagated only through the student network, and the teachers serve only as fixed sources of classification, regression, and structural supervision.

During inference, only the distilled student network is retained. The three teacher models and the auxiliary distillation computations are completely removed, so the proposed multi-teacher distillation framework does not introduce any additional inference cost.

#### 3.3.1. Semantic Classification Teacher

The semantic classification teacher, denoted as Semantic teacher, adopts the standard YOLO11s detector. Since no additional geometric or topological enhancement is introduced, its classification branch provides more stable semantic supervision, and the training process is usually more stable. In the proposed framework, this teacher is not used to provide richer structural priors, but serves as a reliable source of class probabilities and global semantic distributions. The detailed structure is shown in Figure 1.

#### 3.3.2. Lightweight Geometric Regression Teacher

**Figure 5:**
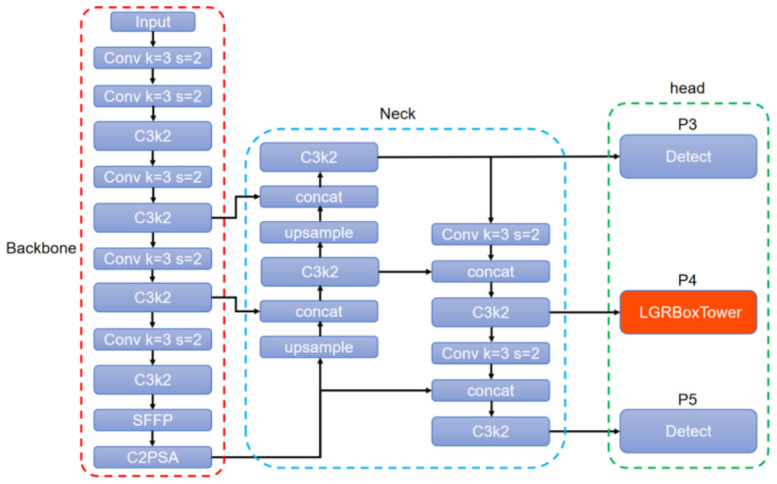
Structure of LGR teacher. The red, blue, and green dashed frames indicate the backbone, neck, and head, respectively.

The lightweight geometric regression teacher, denoted as LGR teacher, keeps the overall architecture of YOLO11n unchanged and only introduces the LGRBoxTower shown in Figure 3 at the P4 stage. In other words, its enhancement is strictly concentrated on P4 geometric regression rather than globally modifying all scales. This design makes the regression supervision closer to the inductive bias of the student and more suitable for transferring local orientation and boundary localization knowledge. Figure 5 shows the architecture of the LGR teacher.

#### 3.3.3. Structural Topology Teacher

**Figure 6:**
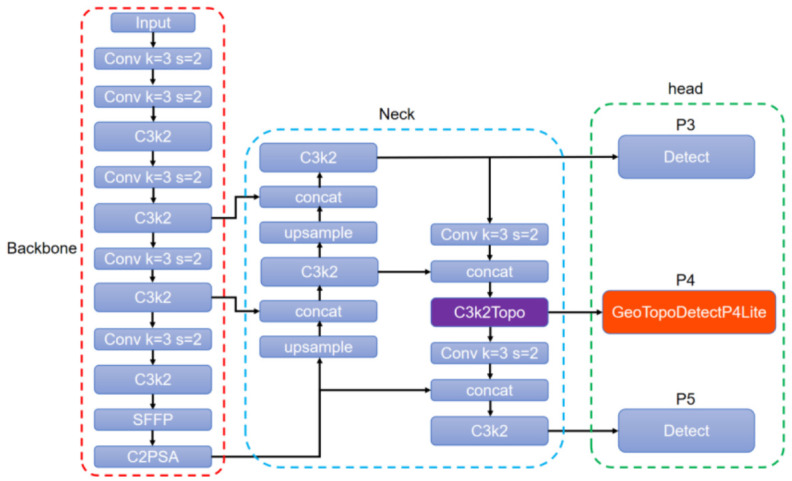
Structure of Topo teacher. The red, blue, and green dashed frames indicate the backbone, neck, and head, respectively.

The structural topology teacher, denoted as Topo teacher, enhances both the neck of P4 and the head of P4. Compared with the LGR teacher, the Topo teacher further emphasizes structural continuity, boundary organization, and the coupling between local geometry and global topology. Figure 6 shows the architecture of the Topo teacher.

**Figure 7:**
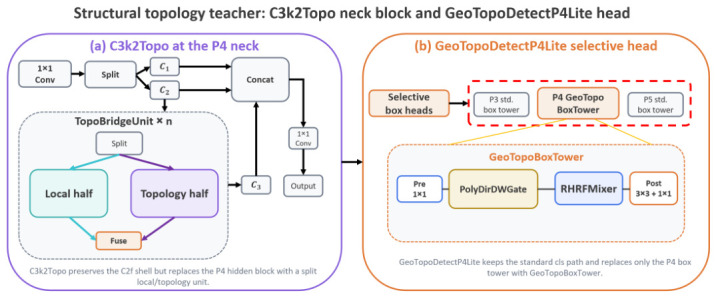
Combined Topo teacher diagram: C3k2Topo at the P4 neck and GeoTopoDetectP4Lite at the P4 box head.

The enhanced structures at the neck end and the head end are jointly illustrated in Figure 7.

As shown in Figure 7, on the neck side, C3k2Topo preserves the external structure of C3k2 and divides the hidden channels into local half and topology half. On the head side, PolyDirDWGate is cascaded with RHRFMixer to implement the fusion of geometric routing and topology. The specific procedure of the C3k2Topo operator is expressed as follows:

(43) $$c_{1},c_{2}=\mathrm{Split}\left({\mathrm{Conv}}_{1\times 1}\left(x\right)\right)$$

(44) $$c_{3}=\mathrm{TopoBridgeUnit}\left(c_{2}\right)$$

(45) $$C3k2\mathrm{Topo}\left(x\right)={\mathrm{Conv}}_{1\times 1}\left(\mathrm{Concat}(c_{1},c_{2},c_{3})\right)$$

TopoBridgeUnit(.) is obtained through the following computation:

(46) $$x_{1},x_{2}=\mathrm{Split}\left(x\right)$$

(47) $$y_{1}=\mathrm{local}\_\mathrm{half}\left(x_{1}\right)={\mathrm{Conv}}_{1\times 1}\left(\mathrm{BN}({\mathrm{DWConv}}_{3\times 3}\left(x_{1}\right))\right)$$

(48) $$x_{1}=x_{1}+\sigma \left(\alpha \right)\left(y_{1}-x_{1}\right)$$

(49) $$x_{2}=\mathrm{Topology}\_\mathrm{half}\left(x_{2}\right)=\mathrm{RHRFMixer}\left(\mathrm{PolyDirDWGate}({\mathrm{Conv}}_{1\times 1}\left(x_{2}\right))\right)$$

(50) $$\mathrm{out}={\mathrm{Conv}}_{1\times 1}\left(\mathrm{Concat}(x_{1},x_{2})\right)$$

σ(α) denotes the residual gating coefficient; the specific computation of the *PolyDirDWGate* operator is as follows:

(51) $$\begin{array}{cc} z & =\mathrm{SiLU}\left(\mathrm{BN}({\mathrm{Conv}}_{1\times 1}\left(x\right))\right);\\ \lambda & =\sigma \left({\mathrm{Conv}}_{1\times 1}\left(z\right)\right) \end{array}$$

(52) $$\begin{array}{cc} w_{1},w_{2},w_{3},w_{4} & =\mathrm{Softmax}\left({\mathrm{Conv}}_{1\times 1}\left(\mathrm{SiLU}(\mathrm{BN}\left({\mathrm{DWConv}}_{3\times 3}\left(z\right)\right))\right)\right);\\ r_{1} & =y_{v}={\mathrm{DWConv}}_{k\times 1}\left(x\right);\\ r_{2} & =y_{h}={\mathrm{DWConv}}_{1\times k}\left(x\right);\\ r_{3} & =y_{d2}=\mathrm{AntiDiagDWConv}\left(x,k=7,\mathrm{band}=3\right);\\ r_{4} & =y_{d1}=\mathrm{MainDiagDWConv}\left(x,k=7,\mathrm{band}=3\right);\\ r_{5} & =y_{sq}={\mathrm{DWConv}}_{3\times 3}\left(x\right) \end{array}$$

(53) $$y=\lambda ⊙r_{5}+\left(1-\lambda \right)\sum_{n=1}^{4}w_{n}⊙r_{n},\;\mathrm{PolyDirDWGate}\left(x\right)={\mathrm{Conv}}_{1\times 1}\left(y\right)$$

where λ denotes the fusion coefficient between the isotropic branch and the directional branches; $w_{1}$, $w_{2}$, $w_{3}$, and $w_{4}$ denote the weights of the vertical, horizontal, anti-diagonal, and main-diagonal branches, respectively. The routing weights $w_{n}$ provide direction-adaptive geometric routing, and the fused output of PolyDirDWGate is further processed by RHRFMixer for topology-aware channel mixing.

For the GeoTopoBoxTower detection head, let the output feature of C3k2Topo be *x*, and the specific computation process is as follows:

(54) $$\mathrm{box}={\mathrm{Conv}}_{1\times 1}\left({\mathrm{Conv}}_{3\times 3}\left(\mathrm{RHRFMixer}(\mathrm{PolyDirDWGate}\left({\mathrm{Conv}}_{1\times 1}\left(x\right)\right))\right)\right)$$

Through the combination of C3k2Topo and GeoTopoBoxTower, the Topo teacher can adaptively select the optimal geometric direction to perceive the boundaries of elongated and inclined objects.

### 3.4. Multi-Teacher Adaptive Weighted Distillation

#### 3.4.1. Branch-Decoupled Teacher Weighting

In the proposed distillation framework, the three teachers do not share a globally fixed weight. Instead, independent teacher weights are computed separately for the classification branch and the regression branch. This design is particularly important in optical remote sensing object detection, because the teacher branch with higher confidence can change with categories, samples, and even training stages. When inference is performed, the framework only inherits the static weights of the student network and therefore does not need to adjust the joint influence of teachers on the student network during training. In the implementation, the teacher quality scores and the corresponding adaptive weights are computed under a no-gradient mode and are detached from the computational graph. Thus, the dynamic weighting mechanism only adjusts the relative contributions of different teacher supervision signals, while the teacher networks themselves remain fixed throughout the student training process. Specifically, the classification reliability is estimated from the foreground classification responses of each teacher, whereas the regression reliability is estimated in an assignment-aware and GT-guided manner using the matched foreground boxes. Both reliability estimates are used only for weighting distillation losses during training and are not involved in inference.

#### 3.4.2. Classification, Box, and DFL Distillation

The distillation objective mainly consists of three components. Classification distillation uses the binary cross-entropy form between the student logits and the sigmoid scores of the dynamically weighted teachers; box distillation first decodes the discrete distributions of the student and teacher into actual bounding boxes, and then applies Smooth L1 supervision; DFL distillation aligns the discrete regression distributions of the student and teacher through KL divergence over foreground locations. Through this joint distillation, the student model learns not only the trend of the teachers’ final outputs but also the shape information of their regression distributions.

#### 3.4.3. Proxy Topology-Assisted Transfer

In addition to the main distillation items, the student network also retains the proxy auxiliary heads during training, i.e., proxy auxiliary outputs, which are used to generate responses related to the centerline and boundary. The distillation block further collects structural auxiliary responses from the topology teacher and aligns them with the student through the squared error. The role of this design is to more smoothly transfer topology-aware structural cues to the student. However, it does not alter the final inference graph, and is therefore more suitable as a secondary training enhancement rather than the core innovation of the method.

**Figure 8:**
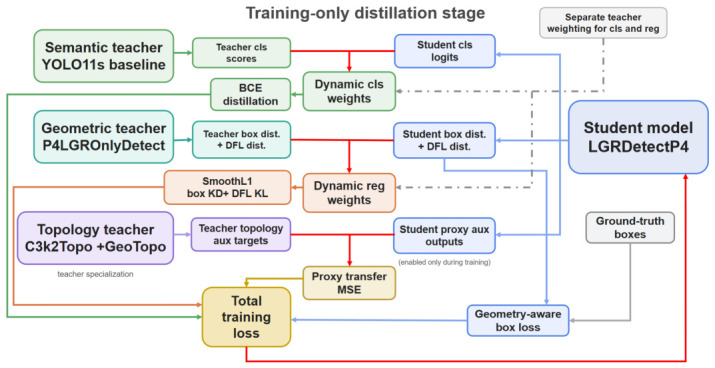
Dynamic multi-teacher distillation with explicit student/teacher paths. Different arrow colors denote different loss and distillation paths, and dashed gray lines indicate the dynamic teacher-weighting paths.

In the section covering distillation, this paper summarizes the multi-teacher adaptive weighted distillation process in Figure 8.

Unlike traditional multi-teacher distillation, which adopts fixed weighting coefficients, the proposed method dynamically estimates the teacher models from two perspectives, namely classification reliability and regression reliability, and recomputes the distillation weights of each teacher network in each training batch. Let the teacher set be denoted as M={Sem,LGR,Topo}, corresponding to the Semantic teacher, LGR teacher, and Topo teacher, respectively. Here, M denotes the set of all teacher models, m∈M denotes one specific teacher model, and n∈M is used as the summation index when normalizing the teacher weights.

(1) The teacher quality estimation for the classification branch and the adaptive weight computation for classification distillation can be obtained through the following procedure:

(55) $$q_{m}^{\mathrm{cls}}={\mathrm{Conf}}_{m}-0.25{\mathrm{Ent}}_{m}$$

(56) $$\alpha_{m}^{\mathrm{cls}}=\frac{\exp(q_{m}^{\mathrm{cls}}/\tau )}{\sum_{n\in M}\exp\left(q_{n}^{\mathrm{cls}}/\tau \right)}$$

For the classification branch, the average maximum classification confidence ${\mathrm{Conf}}_{m}$ and the classification entropy ${\mathrm{Ent}}_{m}$ of the *m*-th teacher over the foreground samples are computed, and the classification quality score $q_{m}^{\mathrm{cls}}$ is constructed. The classification distillation weight $\alpha_{m}^{\mathrm{cls}}$ is then obtained through Softmax normalization with temperature τ. Assume that each teacher *m* outputs classification logits $z_{m}\in R^{B\times A\times N_{\mathrm{cls}}}$, where *B* is the batch size, *A* denotes the number of detection locations, and $N_{\mathrm{cls}}$ is the number of object categories. Then, ${\mathrm{Conf}}_{m}$ and ${\mathrm{Ent}}_{m}$ can be computed as follows:

(57) $${\mathrm{Conf}}_{m}=\frac{1}{\left|\Omega \right|}\sum_{a\in \Omega }\max_{j\in [1,N_{\mathrm{cls}}]}p_{m,a,j},\;p_{m}=\sigma \left(z_{m}\right)$$

(58) $${\mathrm{Ent}}_{m}=\frac{1}{\left|\Omega \right|}\sum_{a\in \Omega }-\frac{1}{\log N_{\mathrm{cls}}}\sum_{j=1}^{N_{\mathrm{cls}}}{\bar{p}}_{m,a,j}\log\left({\bar{p}}_{m,a,j}+\varepsilon \right),\;{\bar{p}}_{m,a,j}=\frac{p_{m,a,j}}{\sum_{k=1}^{N_{\mathrm{cls}}}p_{m,a,k}+\varepsilon }$$

where Ω denotes the foreground sample set and |Ω| denotes the number of foreground samples; $p_{m}$ denotes the classification probability obtained from the teacher logits; $p_{m,a,j}$ denotes the classification probability of the *m*-th teacher for the *j*-th category at the *a*-th foreground sample; ${\bar{p}}_{m,a,j}$ denotes the normalized category distribution; σ(·) denotes the sigmoid activation function; and ε is a small constant used for numerical stability.

(2) The quality evaluation of teachers for the regression branch and the adaptive weight calculation for regression distillation can be obtained through the following computation process:

(59) $$q_{m}^{\mathrm{reg}}=2{\mathrm{IoU}}_{m}+0.25P_{m}^{\max}$$

(60) $$\alpha_{m}^{\mathrm{reg}}=\frac{\exp(q_{m}^{\mathrm{reg}}/\tau )}{\sum_{n\in M}\exp\left(q_{n}^{\mathrm{reg}}/\tau \right)}$$

For the regression branch, the teacher’s bounding box distribution is first decoded into predicted boxes, and the average IoU between the predicted boxes and the target boxes is computed. Meanwhile, the mean of the maximum probabilities in the teacher’s DFL distribution, denoted as $P_{m}^{\max}$, is calculated. The regression quality score $q_{m}^{\mathrm{reg}}$ is then obtained, and the regression distillation weight $\alpha_{m}^{\mathrm{reg}}$ is obtained through Softmax normalization. Let $d_{m}\in R^{B\times A\times 4R}$ be the bounding box distribution logits of the *m*-th teacher, where *R* is the number of discrete bins in DFL, and 4R denotes the discrete distributions in the four bounding box directions. Then, ${\mathrm{IoU}}_{m}$ and $P_{m}^{\max}$ can be computed as follows:

(61) $${\mathrm{IoU}}_{m}=\frac{1}{\left|\Omega \right|}\sum_{a\in \Omega }\mathrm{IoU}\left({\hat{b}}_{m,a},b_{a}^{\mathrm{gt}}\right),\;{\hat{b}}_{m}=\mathrm{Decode}\left(d_{m},\mathrm{anchor}\right)$$

(62) $$P_{m}^{\max}=\frac{1}{4|\Omega |}\sum_{a\in \Omega }\sum_{r=1}^{4}\max_{k\in [1,R]}p_{m,a,r,k},\;p_{m}=\mathrm{Softmax}\left(d_{m}\right)$$

where ${\hat{b}}_{m,a}$ denotes the predicted box decoded from the bounding box distribution of the *m*-th teacher at the *a*-th foreground sample; $b_{a}^{\mathrm{gt}}$ denotes the ground-truth box matched to the *a*-th foreground candidate position after the task-aligned label assignment strategy; Decode(·) denotes the bounding box decoding operation; $p_{m,a,r,k}$ denotes the probability value of the *m*-th teacher at the *a*-th foreground sample, for the distance in the *r*-th bounding box direction, in the *k*-th discrete bin. It should be noted that the regression reliability is GT-guided and is computed only during training based on the assigned foreground samples. No ground-truth boxes, teacher models, or dynamic weighting modules are required during inference.

(3) The classification distillation loss $L_{\mathrm{cls}}^{\mathrm{KD}}$, the decoded bounding box distillation loss $L_{\mathrm{box}}^{\mathrm{KD}}$, the bounding box distribution distillation loss $L_{\mathrm{dfl}}^{\mathrm{KD}}$, and the topology proxy auxiliary distillation loss $L_{\mathrm{proxy}}$ are computed as follows:

(63) $$L_{\mathrm{cls}}^{\mathrm{KD}}=\sum_{m\in M}\alpha_{m}^{\mathrm{cls}}\mathrm{BCE}\left(z_{\mathrm{stu}},\sigma \left(z_{m}\right)\right)$$

(64) $$L_{\mathrm{box}}^{\mathrm{KD}}=\sum_{m\in M}\alpha_{m}^{\mathrm{reg}}\mathrm{SmoothL}1\left({\hat{b}}_{\mathrm{stu}}^{F},{\hat{b}}_{m}^{F}\right)$$

(65) $$L_{\mathrm{dfl}}^{\mathrm{KD}}=\sum_{m\in M}\alpha_{m}^{\mathrm{reg}}\mathrm{KL}\left(p_{m}^{\mathrm{dfl},F}\|p_{\mathrm{stu}}^{\mathrm{dfl},F}\right),\;p_{\mathrm{stu}}^{\mathrm{dfl}}=\mathrm{Softmax}\left(d_{\mathrm{stu}}\right),\;p_{m}^{\mathrm{dfl}}=\mathrm{Softmax}\left(d_{m}\right)$$

(66) $$L_{\mathrm{proxy}}=\sum_{i=1}^{N_{\mathrm{cen}}}\mathrm{MSE}\left(\sigma \left(C_{i}^{\mathrm{stu}}\right),\sigma \left(C_{i}^{\mathrm{topo}}\right)\right)+\sum_{i=1}^{N_{\mathrm{bd}}}\mathrm{MSE}\left(\sigma \left(B_{i}^{\mathrm{stu}}\right),\sigma \left(B_{i}^{\mathrm{topo}}\right)\right)$$

In addition to the topology proxy distillation from the topology teacher, the auxiliary maps of the student are also supervised by GT-derived centerline and boundary targets. The GT-guided auxiliary structural loss is formulated as follows:

(67) $$\begin{array}{cc} L_{\mathrm{aux}}^{\mathrm{GT}}= & \sum_{i=1}^{N_{\mathrm{cen}}}\mathrm{Mean}\left[\left(1+2T_{c,i}^{\mathrm{GT}}\right)\mathrm{BCE}\left(C_{i}^{\mathrm{stu}},T_{c,i}^{\mathrm{GT}}\right)\right]\\ \; & +0.75\sum_{i=1}^{N_{\mathrm{bd}}}\mathrm{Mean}\left[\left(1+2T_{b,i}^{\mathrm{GT}}\right)\mathrm{BCE}\left(B_{i}^{\mathrm{stu}},T_{b,i}^{\mathrm{GT}}\right)\right] \end{array}$$

(68) $$L_{\mathrm{total}}=L_{\det}+\lambda_{\mathrm{cls}}L_{\mathrm{cls}}^{\mathrm{KD}}+\lambda_{\mathrm{box}}L_{\mathrm{box}}^{\mathrm{KD}}+\lambda_{\mathrm{dfl}}L_{\mathrm{dfl}}^{\mathrm{KD}}+\lambda_{\mathrm{proxy}}L_{\mathrm{proxy}}+\lambda_{\mathrm{aux}}L_{\mathrm{aux}}^{\mathrm{GT}}$$

where BCE(·) denotes binary cross-entropy computed from logits unless otherwise specified; SmoothL1(·) denotes the decoded box regression distillation loss; KL(·) denotes the distribution distillation loss; MSE(·) denotes the mean squared error used for auxiliary distillation; and Mean(·) denotes averaging over all elements of the corresponding auxiliary response map. The variable $z_{\mathrm{stu}}$ denotes the classification logits of the student, and $z_{m}$ denotes the classification logits of the *m*-th teacher. The variables ${\hat{b}}_{\mathrm{stu}}^{F}$ and ${\hat{b}}_{m}^{F}$ denote the decoded boxes of the student and the *m*-th teacher over the foreground sample set *F*, respectively. The variables $d_{\mathrm{stu}}$ and $d_{m}$ denote the DFL logits of the student and the *m*-th teacher, respectively, while $p_{\mathrm{stu}}^{\mathrm{dfl},F}$ and $p_{m}^{\mathrm{dfl},F}$ denote their corresponding DFL probability distributions over the foreground sample set. The variables $C_{i}^{\mathrm{stu}}$ and $B_{i}^{\mathrm{stu}}$ denote the *i*-th center auxiliary map and boundary auxiliary map of the student, respectively; $C_{i}^{\mathrm{topo}}$ and $B_{i}^{\mathrm{topo}}$ denote the corresponding center auxiliary map and boundary auxiliary map of the Topo teacher, respectively. The variables $T_{c,i}^{\mathrm{GT}}$ and $T_{b,i}^{\mathrm{GT}}$ denote the centerline target and boundary target generated from the ground-truth boxes for the *i*-th auxiliary map, respectively. $N_{\mathrm{cen}}$ and $N_{\mathrm{bd}}$ denote the numbers of center auxiliary maps and boundary auxiliary maps used for auxiliary supervision, respectively. The factor (1+2T) is used as a positive-region reweighting term for the GT-derived auxiliary target, and the coefficient 0.75 is used to balance the boundary auxiliary loss. $L_{\det}$ denotes the basic detection supervision loss, and $\lambda_{\mathrm{cls}}$, $\lambda_{\mathrm{box}}$, $\lambda_{\mathrm{dfl}}$, $\lambda_{\mathrm{proxy}}$, and $\lambda_{\mathrm{aux}}$ control the contributions of classification distillation, box distillation, DFL distillation, proxy auxiliary distillation, and GT-guided auxiliary structural supervision, respectively.

In the implementation, the loss weights are set to $\lambda_{\mathrm{cls}}=0.40$, $\lambda_{\mathrm{box}}=0.60$, $\lambda_{\mathrm{dfl}}=0.80$, $\lambda_{\mathrm{proxy}}=0.12$, and $\lambda_{\mathrm{aux}}=0.01$. These values correspond to the hyperparameters kd_cls_gain, kd_box_gain, kd_dfl_gain, kd_proxy_gain, and aux_box_gain, respectively.

## 4. Experimental Results and Analysis

### 4.1. Experimental Settings and Evaluation Metrics

This paper mainly conducts structural ablation, distillation mechanism analysis, horizontal comparison, and deployment efficiency evaluation on the DIOR dataset, and further performs supplementary cross-dataset validation on the NWPU VHR-10 dataset. DIOR is a large-scale benchmark dataset for optical remote sensing object detection, containing 20 object categories and covering complex optical remote sensing scenarios such as airports, harbors, roads, bridges, vehicles, ships, and sports fields. According to the official dataset split, the DIOR dataset contains 5862 training images and 5863 validation images. NWPU VHR-10 is also used to evaluate the basic applicability of the proposed method on different optical remote sensing data distributions. It contains 650 target-containing images and 150 background images without targets. This dataset covers 10 categories, including airplane, ship, storage tank, baseball field, tennis court, basketball court, ground track field, harbor, bridge, and vehicle. In this paper, the dataset is randomly divided into training and validation sets at a ratio of 8:2, with approximately 640 images for training and 160 images for validation. All experimental results are reported on the validation set.

All reproducible experiments in this paper are conducted under a unified training and evaluation protocol. The experiments were implemented using Python 3.12.3, PyTorch 2.7.0 with CUDA 12.8, and Ultralytics 8.4.13 on an NVIDIA GeForce RTX 5090 GPU. The input image size is set to 640 × 640, the number of training epochs is 100, the batch size is 16, the optimizer is AdamW, the initial learning rate is $10^{-3}$, the warmup epoch is set to 3, and close mosaic is set to 10. To reduce the randomness introduced by single-run training, this paper repeats training with multiple random seeds in key experiments and reports the mean and standard deviation. The evaluation metrics include mAP50, the number of parameters, GFLOPs, inference latency, FPS, and model file size. Among them, mAP50 is used to measure detection accuracy, the number of parameters and GFLOPs are used to measure the theoretical complexity of the model, and inference latency and FPS are used to evaluate practical deployment efficiency.

For the teacher models used in the distillation stage, all three teachers are trained independently on DIOR before student distillation. To ensure consistency, the Semantic teacher, LGR teacher, and Topo teacher follow the same training configuration as the student model, including 100 training epochs, an input size of 640 × 640, a batch size of 16, the AdamW optimizer, an initial learning rate of 10^−3^, 3 warm-up epochs, and close mosaic set to 10. The same three random seeds are used for teacher training. After training, the checkpoint with the best validation mAP50 among the three runs is selected as the fixed teacher checkpoint for the subsequent distillation process. During distillation, the teacher models are frozen and are used only to generate supervision signals; their parameters are not updated.

Table 1 presents the main training hyperparameters used in the experiments of this paper.

### 4.2. Structural Design Validation

Table 2 presents the experimental results of the main experiments in this paper.

Table 2 presents the results of the structural experiments and the final model Dynamic_3T_KD. It can be observed that, when only a geometric decoupling head is introduced at P4, P4LGROnlyDetect improves mAP50 from 0.80132 to 0.80207, indicating that additional modeling capability concentrated at P4 is effective; however, the number of parameters of this scheme slightly increases to 2.642 M, and GFLOPs becomes 6.601 G, suggesting that strengthening P4 alone is still insufficient to yield an ideal lightweight benefit. In contrast, P35LiteDetect, which only performs lightweight design on P3/P5, reduces the number of parameters to 2.369 M and GFLOPs to 5.521 G, but decreases mAP50 to 0.79758, indicating that simply compressing the high- and low-level detection branches may damage stable localization capability. Compared with the stronger topology-enhanced structure C3k2Topo_Geo, the final student achieves a comparable mAP50, with only a negligible decrease from 0.80363 to 0.80360, while further reducing the number of parameters by approximately 3.63% and GFLOPs by approximately 9.53%. More importantly, the comparison between LGRDetectP4_no_loss and LGRDetectP4 shows that, when only the lightweight P4 geometric head is introduced without the geometric loss, mAP50 is 0.79918; after adding the geometric adaptive box loss, mAP50 recovers to 0.80057. On this basis, the final Dynamic_3T_KD, while maintaining 2.418 M parameters and 5.563 G GFLOPs, improves mAP50 to 0.80360. Compared with YOLO11n_baseline, Dynamic_3T_KD improves mAP50 by 0.23 percentage points, while reducing the number of parameters by approximately 6.8% and GFLOPs by approximately 13.9%; compared with C3k2Topo_Geo, which achieves an mAP50 of 0.80363, the final student further reduces the number of parameters by approximately 3.63% and GFLOPs by approximately 9.53%. This indicates that the optimal approach in this paper is not to simply stack topology modules, but to achieve a better accuracy–complexity improvement through P3/P5 lightweight design, P4 selective geometric modeling, and subsequent distillation recovery.

Although Dynamic_3T_KD has the same student architecture as LGRDetectP4, it shows a higher training time. The reason is that the proposed dynamic multi-teacher distillation introduces additional teacher forward passes, adaptive teacher weighting, and distillation loss computation during training. In contrast, these teacher networks are not used during validation or inference. Only the trained lightweight student detector is retained. Therefore, the proposed method increases the training-stage cost but does not introduce extra inference overhead, which explains why the validation time remains comparable to other lightweight models.

### 4.3. Distillation Mechanism Analysis

Before analyzing different distillation strategies, Table 3 reports the complexity and validation accuracy of the three teachers used for distillation. The Semantic teacher adopts YOLO11s and achieves the highest mAP50, thus providing strong semantic supervision. The LGR and Topo teachers are more compact and focus on geometric regression and topology-aware structural modeling, respectively. Although their overall mAP50 values are lower, they provide complementary regression and structural priors for the lightweight student.

Table 4 reports the per-class AP@0.5 of different distillation strategies under a single representative seed with median performance for detailed category-level analysis, whereas Table 5 reports the three-seed stability comparison. Therefore, the mAP50 values in the per-class table are used mainly for category-level interpretation. From the overall metrics of this representative run, the mAP50 of Dynamic_3T_KD is 0.804, which is higher than those of Semantic-only, Topo-only, Static 3T, and LGR-only. This indicates that the complementary teacher mechanism can be effectively integrated through dynamic weighting. Hereafter, unless otherwise specified, Semantic-only, Topo-only, and LGR-only denote distillation training using only the Semantic teacher, Topo teacher, and LGR teacher, respectively; Static 3T denotes distillation training using statically fixed weights for multiple teachers.

Further observing the per-category results, Dynamic_3T_KD achieves the strictly best AP in 7 categories and tied-best AP in 1 additional category, resulting in best-or-tied-best performance in 8 out of 20 categories. The advantages are mainly concentrated in categories such as Expressway-toll-station, airport, chimney, groundtrackfield, overpass, ship, tenniscourt, and trainstation. These categories usually involve complex backgrounds, elongated structures, densely distributed instances, or ambiguous boundaries, indicating that dynamic weighting can more effectively exploit the semantic stability of the Semantic teacher, the geometric regression capability of the LGR teacher, and the structural continuity prior of the Topo teacher.

Meanwhile, the best-performing categories of single-teacher and static 3T settings do not completely overlap. Topo-only performs best on categories such as Expressway-Service-area, airplane, bridge, storagetank, vehicle, and windmill, and ties with Semantic-only on harbor. LGR-only obtains the best result on golffield. Static 3T is more advantageous on baseballfield, basketballcourt, and dam. Semantic-only performs best on stadium and ties with Dynamic_3T_KD on overpass. This phenomenon of scattered advantageous categories suggests that the complementary information provided by the three teachers is not redundant knowledge but different types of mutual supervision. If single-teacher or fixed-weight fusion is adopted, it can only dominate in local categories; only by allowing the teacher weights to dynamically adjust along classification and regression branches can such complementarity be transformed into a higher overall mAP50.

Considering that the overall improvement of Dynamic_3T_KD over Static 3T is limited, if the comparison is based only on a single run, it may indeed be easily interpreted as random fluctuation. To further verify the stability of dynamic teacher weighting itself, under the condition that the teacher combination and training protocol are kept consistent, this paper repeats the experiments for Static 3T and Dynamic_3T_KD with 3 random seeds, respectively, and reports the mean ± std of mAP50.

As shown in Table 5, Dynamic_3T_KD consistently outperforms Static 3T under 3 random seeds. Its mAP50 increases from 0.8001±0.0004 to 0.8031±0.0003, with an average absolute gain of 0.0030. More importantly, Dynamic_3T_KD outperforms the other model on all 3/3 seeds, indicating that its advantage is not a single lucky run but represents a stable improvement trend maintained in repeated experiments.

#### 4.3.1. Statistical Analysis of Dynamic Teacher Preferences

To further verify, at the mechanism level, whether dynamic teacher weighting truly takes effect, rather than merely behaving as a black-box gain on the final AP result, this paper further conducts offline statistics on the category-level teacher preferences over the validation set. Specifically, under the condition that the student model and the three expert teachers are kept consistent with the previous process, the dynamic teacher weights of the classification branch and regression branch are separately counted, and weighted averaging is performed according to the number of samples in each category, thereby obtaining category-level average teacher weights and dominant teachers.

As shown in Table 6, the classification branch exhibits an obvious preference toward the Semantic teacher: among the 20 categories, the Semantic teacher has the highest average weight in 18 categories, while only storagetank and chimney are dominated by the LGR teacher and the Topo teacher, respectively. This indicates that, at the category-discrimination level, dynamic weighting is more inclined to select the Semantic teacher with more stable semantic supervision, which is consistent with the design motivation of the Semantic teacher in this paper.

In contrast, the regression branch shown in Table 7 presents more obvious category differences: the Topo teacher dominates in 12 categories, the LGR teacher dominates in 5 categories, and the Semantic teacher dominates in only 3 categories. In other words, dynamic teacher selection does not impose a global fixed preference on a particular teacher, but varies with the branch function and category attributes. Therefore, the teacher-preference statistics here provide direct mechanistic evidence for “category–regression-branch decoupled multi-teacher adaptive weighting”.

#### 4.3.2. Attribute-Oriented Group Analysis for Multi-Teacher Complementarity

To further verify whether the proposed teachers provide complementary supervision for different optical remote sensing detection difficulties, this paper conducts an attribute-oriented group analysis in addition to the category-wise AP50 comparison. Specifically, according to the validation annotations and image content, we construct three category groups, including the small-target group, elongated-target group, and complex-background group. These groups are used to evaluate the group-wise mAP50 of different distillation settings, so as to analyze whether each teacher contributes to the corresponding challenging categories.

For the *i*-th ground-truth instance of category *c*, the bounding box is denoted as $b_{i}=\left(x_{i},y_{i},w_{i},h_{i}\right)$, where $w_{i}$ and $h_{i}$ represent the normalized width and height in the YOLO annotation format. The instance-level attributes are defined as follows:

(69) $$\begin{array}{cc} a_{i} & =w_{i}h_{i},\\ r_{i} & =\max\left(\frac{w_{i}}{h_{i}},\frac{h_{i}}{w_{i}}\right),\\ e_{i} & =H\left(\mathrm{Ring}(I,b_{i};s)\right),\;s=1.8. \end{array}$$

In the above formulation, $a_{i}$ denotes the normalized object area and is used to describe object scale. $r_{i}$ denotes the elongation ratio and is used to describe the anisotropy of object shape. $e_{i}$ denotes the gray-level entropy of the outer-ring region around the ground-truth box, which is used to characterize the background complexity near the object. Ring(·) denotes the outer-ring region obtained by expanding the ground-truth box by a scale factor *s* and then removing the original box region. H(·) denotes the Shannon entropy of the gray-level distribution in this region.

At the category level, the median value of all instances belonging to category *c* is used as the category-level attribute statistic:

(70) $$\begin{array}{cc} A_{c} & ={\mathrm{median}}_{i\in \Omega_{c}}\left(a_{i}\right),\\ R_{c} & ={\mathrm{median}}_{i\in \Omega_{c}}\left(r_{i}\right),\\ E_{c} & ={\mathrm{median}}_{i\in \Omega_{c}}\left(e_{i}\right), \end{array}$$

where $\Omega_{c}$ denotes the set of instances belonging to category *c*. $A_{c}$, $R_{c}$, and $E_{c}$ correspond to the category-level scale statistic, elongation statistic, and background-complexity statistic, respectively.

Let the complete category set be denoted as C, and we set K=5 to select the most representative categories for each attribute group. The three attribute-oriented category groups are defined as follows:

(71) $$\begin{array}{cc} G_{\mathrm{small}} & ={\mathrm{TopK}}_{↑}\left(\left\{A_{c}|c\in C\right\},K\right),\\ G_{\mathrm{elongated}} & ={\mathrm{TopK}}_{↓}\left(\left\{R_{c}|c\in C\right\},K\right),\\ G_{\mathrm{complex}} & ={\mathrm{TopK}}_{↓}\left(\left\{E_{c}|c\in C\right\},K\right). \end{array}$$

Here, ${\mathrm{TopK}}_{↑}$ selects the top *K* categories in ascending order, while ${\mathrm{TopK}}_{↓}$ selects the top *K* categories in descending order. Thus, $G_{\mathrm{small}}$, $G_{\mathrm{elongated}}$, and $G_{\mathrm{complex}}$ correspond to the small-target group, elongated-target group, and complex-background group, respectively. It should be noted that the three groups are constructed according to independent attributes rather than mutually exclusive labels. Therefore, one category may appear in multiple groups when it simultaneously exhibits multiple challenging attributes.

For model *m* on an attribute group G, the group-wise mAP50 is computed as follows:

(72) $$\begin{array}{cc} {\mathrm{GroupAP}}_{50}^{\left(m\right)}\left(G\right) & =\frac{1}{\left|G\right|}\sum_{c\in G}{\mathrm{AP}}_{50,c}^{\left(m\right)},\\ \Delta {\mathrm{GroupAP}}_{50}^{\left(m_{1}-m_{2}\right)}\left(G\right) & ={\mathrm{GroupAP}}_{50}^{\left(m_{1}\right)}\left(G\right)-{\mathrm{GroupAP}}_{50}^{\left(m_{2}\right)}\left(G\right). \end{array}$$

The first equation measures the average localization and classification performance of model *m* on a specific attribute group, while the second equation explicitly measures the absolute AP50 gain between two models on the same group. The definitions of the attribute-oriented category groups and the compared distillation settings are summarized in Table 8.

As shown in Table 9, different single-teacher variants exhibit distinct advantages on different attribute-oriented groups. The Topo. T variant achieves the best AP50 on the small-target group, indicating that structural and contextual topology is helpful for detecting categories with weak object appearance. The LGR. T variant performs best on the elongated-target group, which confirms that geometric regression supervision is more effective for categories with large aspect-ratio variations and higher localization difficulty. On the complex-background group, Sem. T obtains the highest AP50, suggesting that enhanced semantic discrimination is beneficial for suppressing background interference and category ambiguity.

It is also worth noting that Static 3T does not consistently improve over the baseline. This indicates that directly combining multiple teachers with fixed weights may introduce redundant or conflicting supervision. In contrast, Dynamic 3T consistently outperforms both Baseline and Static 3T on all three attribute groups. Specifically, Dynamic 3T improves AP50 from 0.7353 to 0.7434 on the small-target group, from 0.8008 to 0.8106 on the elongated-target group, and from 0.7882 to 0.8049 on the complex-background group. The mAP50 over the three groups is also improved from 0.7748 to 0.7863 compared with Baseline, and from 0.7732 to 0.7863 compared with Static 3T.

These results demonstrate that the proposed dynamic multi-teacher distillation does not simply pursue the best performance on a single attribute group. Instead, it provides more balanced and robust improvements across heterogeneous and challenging scenarios. This observation is consistent with the motivation of introducing semantic classification, geometric regression, and structural topology teachers, and further verifies the necessity of dynamic heterogeneous teacher integration.

#### 4.3.3. Qualitative Visualization Comparison and Typical Failure Case Analysis

**Figure 9:**
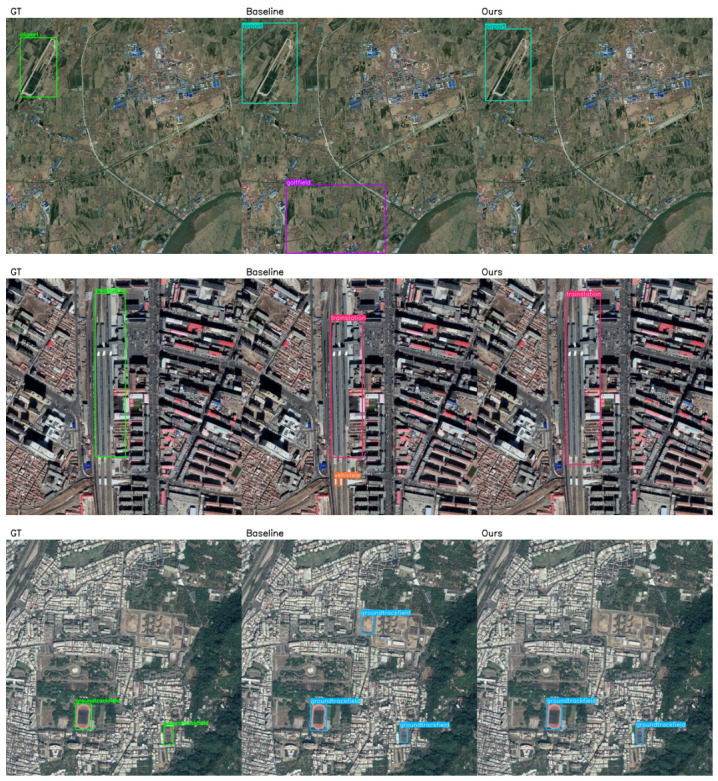
Qualitative comparison among ground truth, YOLO11n baseline, and the proposed method on representative DIOR images. The columns labeled GT, Baseline, and Ours denote the ground-truth annotations, YOLO11n baseline predictions, and predictions of the proposed method, respectively. The frame colors are automatically assigned according to object categories by the visualization tool, and the category names are shown next to the corresponding boxes.

**Figure 10:**
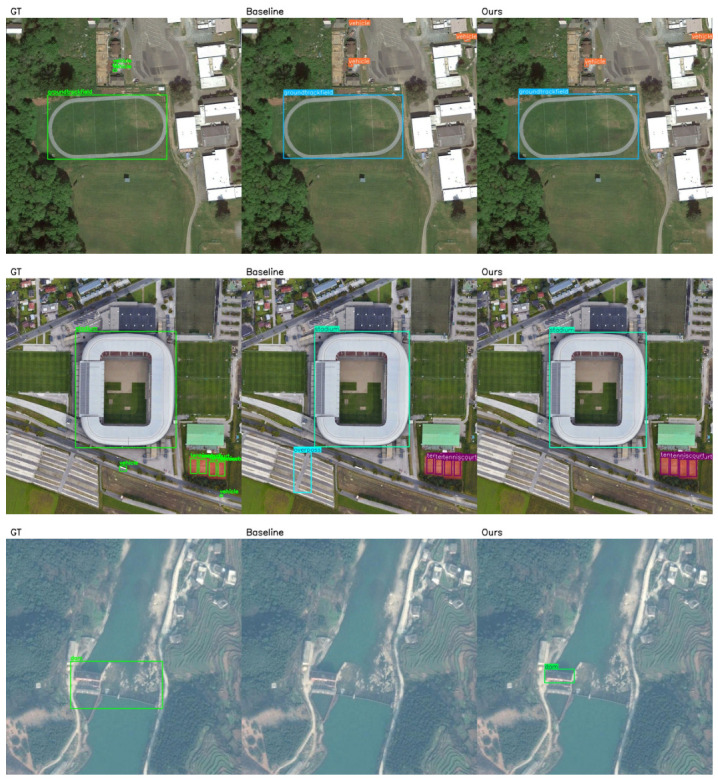
Typical failure cases of the proposed method in complex optical remote sensing scenes. The frame colors are automatically assigned according to object categories by the visualization tool, and the category names are shown next to the corresponding boxes.

To further verify the source of the practical improvement provided by the proposed method in difficult categories and complex scenarios, this paper additionally provides qualitative visualization comparison results of GT, Baseline, and Ours. As shown in Figure 9 and Figure 10, consistent with the previous classification AP analysis and teacher-preference statistics, these visualization examples are selected from categories and complex backgrounds for which the target scale, instance morphology, and background interference are more representative, and are used to intuitively demonstrate the specific effects of multi-teacher adaptive distillation on target recall, false-positive suppression, and localization stability.

As shown in Figure 9, the proposed method exhibits more stable detection behavior than the baseline in several representative scenarios. First, in the airport scenario, the baseline produces an additional false detection of a golf field, whereas our method can preserve the main targets and suppress irrelevant category responses, indicating that the proposed method has stronger background suppression capability in large and complex scenes. Second, in another train station scenario, the baseline has an additional false detection of a vehicle, while the prediction results of our method are cleaner, further demonstrating that multi-teacher adaptive distillation helps reduce false alarms under complex backgrounds. Finally, in the groundtrackfield sample, both the baseline and our method successfully detect the two annotated groundtrackfield targets. Compared with the baseline, our method produces more consistent predictions without introducing additional false positives in the surrounding urban and vegetation areas. This result suggests that the proposed method can better maintain target recall and localization stability for small-scale sports-field objects under complex backgrounds, especially when the targets are embedded in dense buildings, roads, and vegetation.

Meanwhile, Figure 10 presents several typical failure cases to avoid drawing one-sided conclusions from successful examples alone. In the first ground track field sample, both the baseline and our method can detect the main ground track field target, but they also produce false-positive vehicle predictions in the surrounding area. This indicates that the proposed method does not completely eliminate false alarms when small objects are embedded in complex background regions. One possible reason is that the local textures of parking lots, roofs, shadows, and narrow road structures are visually similar to small vehicles from the overhead perspective, which leads to semantic ambiguity between true vehicles and background patterns. In addition, the small object scale makes the discriminative cues weak, so the lightweight student model may still inherit uncertain responses from different teachers.

In the second stadium sample, both methods correctly locate the main stadium, but incorrect predictions remain around the neighboring sports facilities and road-like structures. The baseline misclassifies an elongated road structure as an overpass, while our method still produces false responses on adjacent tennis-court regions. This failure suggests that category confusion may occur when different man-made objects share similar geometric layouts, regular boundaries, and repetitive line patterns in remote sensing images. Although multi-teacher adaptive distillation improves the overall detection stability, the teachers may provide inconsistent supervision for visually similar categories such as stadium, tennis court, overpass, and other sports-field structures. As a result, the student detector may learn ambiguous category boundaries and produce false positives in neighboring regions.

In the third dam sample, the baseline fails to detect the target, while our method recovers only a partial dam region. However, the predicted bounding box is significantly smaller than the ground-truth annotation, indicating that the proposed method still has limitations in complete boundary localization for elongated and low-contrast objects. This is mainly because the dam is visually connected with surrounding water, roads, mountains, and construction areas, and its complete spatial extent is difficult to distinguish from the background. Compared with objects that have closed and regular shapes, such as stadiums or ground track fields, dams usually have weaker texture contrast and less salient geometric boundaries. Therefore, the lightweight student model may obtain sufficient semantic activation to identify part of the target, but its geometric representation is still insufficient for accurate full-object localization.

These failure cases indicate that, although the proposed multi-teacher adaptive distillation strategy improves the discrimination and localization ability of the lightweight detector in many complex scenes, there remains room for further optimization. The remaining errors are mainly related to semantic ambiguity among visually similar categories, insufficient geometric representation for objects with weak or elongated boundaries, complex background interference, and possible inconsistency among teacher predictions. Future work will further explore boundary-aware distillation, uncertainty-guided teacher weighting, and category-specific geometric constraints to improve the robustness of lightweight remote sensing object detectors in highly ambiguous scenarios.

### 4.4. Horizontal Comparison with Existing Lightweight Methods

To evaluate the external competitiveness of the proposed method in lightweight optical remote sensing object detection, Table 8 compares Dynamic_3T_KD with several existing lightweight detectors. It should be noted that the results in the table come from two sources: one consists of results directly reported in the original papers or public materials, and the other consists of reproduced experimental results obtained under the unified DIOR training and evaluation protocol in this paper. For improved methods whose authors do not release complete source code or provide sufficient training details, this paper retains only the results reported in the original papers as external references, rather than regarding them as strictly fair comparisons under identical conditions. For official publicly available baseline models that can be directly trained, this paper additionally provides reproduced experimental results under a unified protocol to improve the interpretability of the comparison.

As shown in Table 10, Dynamic_3T_KD (Ours) achieves an mAP50 of 0.8036 with 2.418 M parameters and 5.56 G GFLOPs. Compared with YOLO11n_baseline (Ours), which has 2.594 M parameters, 6.46 G GFLOPs, and an mAP50 of 0.8013, the proposed method improves accuracy by 0.23 percentage points while reducing the number of parameters by approximately 6.8% and GFLOPs by approximately 13.9%. Considering that YOLO11n itself is already a compact nano baseline designed for deployment scenarios, this improvement of “further compression with accuracy improvement rather than degradation” better demonstrates the effectiveness of the proposed method in accuracy–efficiency optimization for lightweight models.

For lightweight models reported in the literature, the proposed method is approximately 0.1 percentage points lower than YOLOv5s in terms of mAP50, while reducing the number of parameters and GFLOPs by approximately 65.6% and 64.8%, respectively. Compared with Improved YOLOv5s, although the mAP50 is 1.3 percentage points lower, the number of parameters and GFLOPs are only approximately 25.0% and 30.2% of those of Improved YOLOv5s, respectively. Meanwhile, compared with YOLOv8n and YOLOv8n-CTAM, the proposed method achieves mAP50 improvements of 5.6 and 3.8 percentage points, respectively, while maintaining a better or comparable level of complexity. It should be noted that most of these improved methods do not release complete source code. Therefore, this paper can only cite the results reported in the original papers, rather than strictly reproducing them under a fully unified experimental protocol. Accordingly, this comparison is more appropriately regarded as a reference for external competitiveness rather than an absolutely fair comparison under identical conditions.

For publicly trainable official baseline models, this paper further provides reproduced experimental results under a unified protocol. It is worth noting that the reproduced results of YOLOv5s differ from the parameter counts reported on the official website or in the original public materials, indicating that different code versions, default architectural implementations, and training configurations can affect the final statistical results. Therefore, simply citing public tables without unified reproduction may not fully reflect the actual performance of models on DIOR. Based on the same consideration, this paper also supplements the results of YOLOv8n(reproduced) and YOLO26n(reproduced). Among them, YOLO26, as a nano-level model from the same Ultralytics codebase, is included as an additional official lightweight reference baseline. According to the results in Table 8, YOLO26n(reproduced) achieves an mAP50 of 0.802 with 2.512 M parameters and 5.81 G GFLOPs, whereas the proposed model achieves 2.418 M parameters, 5.56 G GFLOPs, and an mAP50 of 0.803, respectively, thus achieving slightly higher accuracy with lower complexity. This indicates that the advantage of the proposed method does not lie in simply pursuing the highest absolute accuracy, but rather in further achieving a better accuracy–efficiency trade-off on a compact nano-level baseline.

Overall, the competitiveness of the proposed method in horizontal comparisons is mainly reflected in two aspects: first, when compared with larger-scale lightweight models, it can maintain comparable detection accuracy with significantly fewer parameters and lower GFLOPs; second, when compared with current representative official nano-level models, it can still maintain slightly better detection performance with lower complexity. This demonstrates that the proposed “lightweight P4 geometric modeling + multi-teacher adaptive distillation” does not simply compress the model to a smaller size. Instead, under a limited complexity budget, it selectively preserves geometric representations that are more relevant to DIOR, and restores the capabilities most vulnerable to compression through complementary teachers, thereby achieving simultaneous optimization of detection accuracy, model complexity, and inference efficiency with greater practical value.

### 4.5. Comparison with Representative Knowledge Distillation Baselines

We further compare the proposed method with recent knowledge distillation strategies. Specifically, we implement two representative single-teacher distillation baselines on the attribute-oriented groups, namely Feature KD and Masked KD. Feature KD follows the common feature-based distillation paradigm, which transfers intermediate feature representations from the teacher detector to the student detector. Masked KD follows the mask-based distillation paradigm, which applies spatial masks to emphasize informative regions during feature imitation. It should be noted that these two baselines are implemented as representative feature-based and mask-based distillation strategies rather than exact reproductions of the above methods.

For a fair comparison, both Feature KD and Masked KD adopt the same teacher model. According to the overall validation performance in Table 9, the topology-teacher-only distillation setting achieves the best mAP50 among the three single-teacher variants. Therefore, the topology teacher is selected as the teacher model for both Feature KD and Masked KD. This setting avoids performance variations caused by different teacher capacities and allows the comparison to focus on the effectiveness of the distillation strategy itself. In contrast, Static 3T and Dynamic 3T use the three task-specific teachers, including the semantic classification teacher, the local geometric regression teacher, and the structural topology teacher. Static 3T fuses the three teachers with fixed weights, whereas Dynamic 3T adaptively integrates heterogeneous teacher knowledge according to the proposed teacher-weighting mechanism.

As shown in Table 11, both Feature KD and Masked KD improve the mAP50 over the baseline, which confirms that conventional single-teacher distillation can provide useful supervision for the student detector. Specifically, Feature KD improves the mAP50 from 0.7748 to 0.7789, while Masked KD further improves it to 0.7842. This result indicates that spatially selective distillation is more effective than plain feature imitation in optical remote sensing object detection, especially when the targets are small or surrounded by complex background interference.

On the small-target group, Masked KD achieves the highest AP50 of 0.7513. This is reasonable because the topology teacher is the strongest single teacher on the small-target group, and the masking mechanism further emphasizes informative object-related regions while suppressing less useful background areas. Therefore, Masked KD is particularly beneficial for small objects whose feature responses are easily overwhelmed by large background regions. However, its advantage is not consistent across all attribute groups. On the elongated-target group, Masked KD obtains only 0.8033, which is much lower than Dynamic 3T with 0.8106. This is because elongated objects are more sensitive to bounding-box geometry and localization accuracy, for which the local geometric regression teacher provides more suitable supervision than the topology teacher alone.

On the complex-background group, Dynamic 3T achieves 0.8049, outperforming Feature KD and Masked KD by 0.0163 and 0.0068 AP50, respectively. This suggests that complex-background categories require not only topology-aware contextual information but also semantic discrimination to suppress background ambiguity. Since single-teacher Feature KD and Masked KD only rely on the topology teacher, they cannot fully exploit the complementary semantic and geometric cues provided by the other teachers. In contrast, Dynamic 3T adaptively integrates heterogeneous knowledge from the semantic, geometric, and topology teachers, leading to more balanced improvements across different challenging scenarios.

It is also worth noting that Static 3T performs slightly worse than the baseline on all three attribute groups. This result indicates that simply increasing the number of teachers does not necessarily lead to better distillation. Without adaptive teacher weighting, heterogeneous supervision signals may become redundant or even conflicting. In contrast, Dynamic 3T consistently outperforms Static 3T on all three groups and improves the mAP50 from 0.7732 to 0.7863. This verifies that the performance gain of the proposed method comes from adaptive heterogeneous teacher integration rather than from naively combining multiple teachers.

Overall, although Masked KD achieves the best result on the small-target group, Dynamic 3T obtains the highest average AP50 among all compared methods and performs best on the elongated-target and complex-background groups. These results demonstrate that the proposed method provides a more robust and balanced distillation framework for different optical remote sensing detection difficulties. The comparison also clarifies the difference between our method and representative single-teacher KD methods: Feature KD and Masked KD mainly improve how knowledge is transferred from a single teacher, whereas the proposed Dynamic 3T focuses on adaptively selecting and integrating complementary knowledge from multiple task-specific teachers. Therefore, this experiment further validates the necessity and effectiveness of dynamic heterogeneous multi-teacher distillation.

#### Comparison with Existing Knowledge Distillation Methods

To further clarify the difference between the proposed method and existing knowledge-distillation-based object detection methods, we compare Dynamic 3T with several representative studies, including dual masking feature distillation, spatial-aware adaptive masking distillation, dynamic channel-wise spatial feature distillation, dynamic knowledge distillation for remote sensing imagery, and adaptive knowledge distillation for lightweight remote sensing object detectors [20,21,22,29,30]. Although these methods share the general teacher–student distillation paradigm, their objectives and knowledge-transfer mechanisms are substantially different from those of the proposed framework.

DMKD improves feature-based knowledge distillation by introducing dual masking augmentation, which considers both spatially important regions and channel-wise informative clues during feature reconstruction [21]. SAMKD further explores spatial-aware adaptive masking and performs fine-grained feature distillation across different scales to improve the effectiveness of masked knowledge transfer [22]. These methods mainly focus on how to design more effective masking strategies for feature imitation. In contrast, the proposed Dynamic 3T focuses on what types of teacher knowledge should be transferred and how heterogeneous knowledge from multiple teachers should be adaptively integrated. Specifically, our framework decomposes teacher supervision into semantic classification knowledge, local geometric regression knowledge, and structural topology knowledge, and then dynamically balances their contributions during student training.

DCSF-KD enhances feature transfer by dynamically modeling channel-wise and spatial knowledge during distillation [20]. This method improves the representation transfer between a teacher and a student by exploiting spatial and channel information in feature maps. By comparison, Dynamic 3T is not limited to channel-spatial feature imitation within a single teacher–student pair. Instead, it introduces three task-specific teachers to provide complementary supervision from semantic, geometric, and topological perspectives, which is more suitable for optical remote sensing object detection with small objects, elongated shapes, dense layouts, complex backgrounds, and large variations in scale.

Dynamic knowledge distillation for remote sensing imagery aims to train efficient and accurate detectors by discovering valuable regions for feature imitation, selecting useful instances, and handling hard samples during distillation [29]. Adaptive knowledge distillation for lightweight remote sensing object detectors further improves lightweight detector optimization by transferring multi-scale core features and using stricter regression supervision [30]. These methods are closely related to remote sensing object detection and demonstrate the effectiveness of adaptive distillation in complex remote sensing scenes. However, they mainly focus on improving the distillation process for a single student model through region selection, instance selection, multi-scale feature imitation, or regression supervision. In contrast, the proposed Dynamic 3T explicitly constructs heterogeneous teacher roles and dynamically integrates their complementary knowledge according to different detection difficulties.

In summary, existing knowledge distillation methods mainly improve specific aspects of knowledge transfer, such as masked feature reconstruction, spatial-aware adaptive masking, channel-spatial feature imitation, dynamic region or instance selection, and adaptive supervision for lightweight remote sensing detectors. These methods are valuable for enhancing distillation effectiveness under their respective settings. However, they generally focus on a particular form of feature transfer or a specific optimization objective. In contrast, the proposed Dynamic 3T performs adaptive heterogeneous multi-teacher knowledge integration. The semantic teacher enhances category discrimination, the geometric teacher improves localization and shape-related regression, and the topology teacher provides contextual structural knowledge. The dynamic teacher-weighting mechanism further avoids the redundancy or conflict that may arise from fixed multi-teacher fusion, leading to more balanced improvements across different optical remote sensing detection difficulties.

### 4.6. Supplementary Validation on NWPU VHR-10

To further investigate the applicability of the proposed method under different optical remote sensing data distributions, this paper conducts supplementary validation on an additional optical remote sensing dataset, NWPUVHR-10. It should be noted that the purpose of this experiment is to examine whether the proposed method can remain basically effective beyond DIOR, rather than extending the cross-dataset evaluation into a complete ablation study on the same scale as the main experiments. Therefore, this section retains the comparison among the YOLO11nbaseline, the lightweight student structure LGRDetectP4, the three-teacher static weight distillation Static3T, and the final model Dynamic_3T_KD. Under the same training settings, experiments are repeated with 3 random seeds to report the mean and standard deviation, thereby reducing the influence of randomness from a single run.

Table 12 reports the experimental results on NWPU VHR-10. It can be observed that the average mAP50 of the baseline is 0.90716, whereas the final model Dynamic_3T_KD improves it to 0.91502, with an absolute gain of +0.00786. Meanwhile, the lightweight student structure also achieves an average mAP50 of 0.90883, showing a slight improvement over the baseline. In contrast, the average mAP50 of Static3T is 0.89964, which is lower than that of the baseline. This phenomenon indicates that, in the cross-dataset scenario, the proposed structural design itself exhibits a certain degree of transferability, while multi-teacher adaptive distillation is more conducive than static teacher weighting to further translating such structural advantages into stable performance gains.

It should also be noted that the standard deviation of Dynamic_3T_KD on NWPU VHR-10 is 0.01371, which is higher than the 0.00555 of the baseline. This suggests that, under the current experimental scale, although the proposed method maintains a leading average performance over 3 seeds, its cross-dataset performance still exhibits certain fluctuations. Therefore, this paper tends to interpret this result as follows: the proposed method shows a positive transfer trend and a certain degree of cross-dataset applicability on another optical remote sensing object detection dataset, rather than claiming that its generalization advantage has been fully verified by large-scale cross-dataset benchmarks.

Overall, the supplementary experiments on NWPU VHR-10 provide two additional pieces of evidence for the proposed method: first, the lightweight P4 geometric modeling student constructed in this paper is not effective only on the single DIOR dataset; second, multi-teacher adaptive distillation can still provide a more favorable performance trend than static teacher weighting in cross-dataset scenarios. Combined with the structural ablation, dynamic weight stability analysis, and teacher-preference statistics on DIOR presented above, the advantages of the proposed method can be considered not to rely entirely on accidental adaptation to a specific dataset, but to possess a certain degree of transferability across different optical remote sensing scenarios.

### 4.7. Deployment and Inference Efficiency Analysis

In addition to offline accuracy and complexity metrics, the actual inference performance in the deployment environment also determines whether a lightweight solution has practical application value. To this end, this paper further evaluates the deployment efficiency of the YOLO11n baseline and the proposed final model under three deployment formats, namely PT, ONNX, and TensorRT FP32 static. Specifically, the best-performing checkpoints of the baseline model and the proposed Dynamic_3T_KD model are first exported into the corresponding deployment formats. Then, all exported models are evaluated on the same DIOR validation set under the same hardware and inference settings.

All speed measurements are conducted on an RTX 4060 GPU with an input size of 640×640. The PT format is evaluated using the PyTorch backend, while the ONNX and TensorRT formats are evaluated using their corresponding deployment runtimes after model export. For the TensorRT setting, a static-shape FP32 engine is used, and neither FP16 nor INT8 quantization is enabled. The reported latency follows the timing output of the same inference interface for all compared models and excludes qualitative visualization operations such as drawing bounding boxes, concatenating images, or saving visualized results. Therefore, the deployment results are independent of the visualization scripts used for Figure 9 and Figure 10. All models are evaluated under the same software environment, including Ultralytics 8.4.13, Python 3.12.6, and PyTorch 2.5.1 with CUDA 12.1, on an NVIDIA GeForce RTX 4060 Laptop GPU, to ensure that the relative latency and FPS comparisons are not affected by hardware or runtime differences.

As shown in Table 13, the proposed model exhibits consistent deployment advantages across all three deployment formats. In the PT format, the latency decreases from 22.945 ms to 16.332 ms, corresponding to a reduction of approximately 28.8%, while the FPS increases from 43.58 to 61.23, yielding an improvement of approximately 40.5%. In the ONNX format, the latency decreases from 34.977 ms to 15.517 ms, corresponding to a reduction of approximately 55.6%, while the FPS increases from 28.59 to 64.45, yielding an improvement of approximately 125.4%. In the TensorRT FP32 static format, the latency further decreases from 22.998 ms to 12.950 ms, corresponding to a reduction of approximately 43.7%, while the FPS increases from 43.48 to 77.22, yielding an improvement of approximately 77.6%.

Meanwhile, the model file size is reduced by approximately 5.2%, 6.2%, and 10.6% in the PT, ONNX, and TensorRT formats, respectively. In terms of hardware resource usage, the proposed model maintains comparable average GPU utilization across different deployment formats. Specifically, compared with the baseline model, the average GPU utilization changes by only −0.16, +1.72, and +1.32 percentage points under the PT, ONNX, and TensorRT formats, respectively. This indicates that the proposed model achieves higher inference throughput at a comparable level of GPU utilization, rather than relying on substantially higher GPU resource occupation.

It is also observed that the peak GPU memory usage remains unchanged between the baseline and the proposed model within each deployment format. This phenomenon does not contradict the reduction in model size. The reported peak GPU memory usage is measured at the device level during inference, and therefore includes not only the model parameters but also the CUDA context, runtime initialization overhead, memory caching, inference-framework buffers, input/output tensors, and intermediate feature tensors. For lightweight detectors such as YOLO11n, the parameter memory accounts for only a small fraction of the total observed GPU memory, whereas the runtime and framework-related memory overhead can dominate the peak device memory. As a result, a reduction of several megabytes in model size may not lead to an observable decrease in the total peak GPU memory usage, especially with a batch size of 1 and a fixed input resolution. Therefore, the identical peak GPU memory values mainly indicate that the proposed model does not introduce additional GPU memory overhead, while its efficiency gain is primarily reflected in lower latency, higher FPS, and smaller model size.

These results indicate that the proposed lightweight structural design not only reduces theoretical complexity, but can also be effectively converted into practical inference acceleration after deployment-format export. Since all deployment models are exported from the best-performing checkpoints and evaluated on the same DIOR validation set, hardware platform, batch size, and input settings, the relative speed improvement mainly reflects the reduction in model complexity and the streamlined regression-head design of the proposed student detector. From the comparison among different runtime formats, the acceleration achieved under ONNX and TensorRT is generally greater than that under PT, suggesting that the proposed structural simplification can be effectively reflected in the deployed execution graph, rather than merely appearing lightweight within the PyTorch training/inference framework. Even on TensorRT, where low-level inference optimization has already been extensively performed, the proposed model still maintains a clear reduction in latency and an increase in throughput, demonstrating its practical advantages in efficient inference environments.

## 5. Conclusions

This paper addresses the challenge that accuracy, complexity, and deployment efficiency are difficult to balance in optical remote sensing object detection, and proposes a lightweight geometrically decoupled student network together with a multi-teacher adaptive weighting distillation framework. While maintaining a compact overall architecture, the proposed method introduces scale-aware branch designs and a geometry-oriented regression structure to provide more suitable inductive biases for elongated objects, direction-sensitive objects, and objects in complex backgrounds. The lightweight structural design reduces computational complexity with only a minor accuracy degradation, while the proposed dynamic multi-teacher distillation further compensates for this degradation and improves the final detection accuracy.

In terms of the training strategy, this paper further constructs a multi-teacher system with different knowledge emphases and designs a dynamic weighting distillation mechanism, enabling the student model to adaptively absorb more valuable teacher information according to the different requirements of classification and regression tasks. Experimental results show that the proposed method can steadily improve detection performance while preserving model lightweightness, and achieves better inference efficiency in various deployment environments, demonstrating its strong potential for practical applications. However, the current method still exhibits certain fluctuations when dealing with objects with ambiguous boundaries and cross-dataset transfer. In future work, stronger orientation modeling mechanisms, more fine-grained distillation constraints, and broader data validation can be further incorporated to continuously improve the generalization ability and robustness of the model in complex remote sensing scenarios.

## Figures and Tables

**Table 1:** Training hyperparameter settings for experiments.

Hyperparameter	Value
epochs	100
batch	16
workers	8
imgsz	640
device	0
cache	disk
optimizer	AdamW
warmup_epochs	3
close_mosaic	10
lr0	$1\times 10^{-3}$
seed	1, 2, 3, 4, 5
deterministic	True

*Note:* The number of seeds varies across experiments due to computational cost and is specified in each corresponding table.

**Table 2:** Comparison of different structural designs and the final model on DIOR.

Method	mAP50	Params (M)	Param Ratio (%)	GFLOPs	GFLOPs Ratio (%)	mAP50 Std	Train (h)	Val (h)
YOLO11n_baseline	0.80132	2.594	100.00	6.461	100.00	0.00118	1.308	0.681
P4LGROnlyDetect	0.80207	2.642	101.85	6.601	102.16	0.00028	1.337	0.698
P35LiteDetect	0.79758	2.369	91.32	5.521	85.45	0.00328	1.253	0.637
only_C3k2Topo	0.80109	2.556	98.53	6.325	97.89	0.00289	1.302	0.678
only_Geo	0.80315	2.547	98.18	6.284	97.26	0.00073	1.302	0.677
C3k2Topo_Geo_no_loss	0.80272	2.509	96.72	6.149	95.17	0.00118	1.294	0.676
C3k2Topo_Geo	0.80363	2.509	96.72	6.149	95.17	0.00153	1.293	0.676
LGRDetectP4_no_loss	0.79918	2.418	93.21	5.563	86.10	0.00111	1.257	0.641
LGRDetectP4	0.80057	2.418	93.21	5.563	86.10	0.00095	1.257	0.640
**Dynamic_3T_KD**	**0.80360**	**2.418**	**93.21**	**5.563**	**86.10**	**0.00151**	**1.379**	**0.639**

*Note:* Results are averaged over five random seeds. Train and validation times denote the average time consumed per epoch on the training and validation sets, respectively. Bold values indicate the results of the final model.

**Table 3:** Complexity and validation accuracy of the three teacher models used for distillation on DIOR.

Teacher	Architecture	Params (M)	GFLOPs	mAP50
Semantic teacher	YOLO11s	9.436	21.589	0.8270
LGR teacher	YOLO11n + P4 LGR	2.642	6.601	0.8024
Topo teacher	P4 topology-enhanced	2.509	6.149	0.8058

**Table 4:** Per-class AP@0.5 comparison of different distillation strategies under the same seed setting on DIOR.

Class	Semantic	Topology	LGR	Static 3T	Dynamic 3T-KD
Expressway-Service-area	0.747	**0.756**	0.744	0.750	0.742
Expressway-toll-station	0.664	0.654	0.652	0.647	**0.678**
airplane	0.941	**0.945**	0.925	0.932	0.931
airport	0.857	0.852	0.863	0.842	**0.867**
baseballfield	0.936	0.942	0.941	**0.947**	0.943
basketballcourt	0.836	0.837	0.828	**0.855**	0.841
bridge	0.509	**0.513**	0.488	0.507	0.502
chimney	0.902	0.908	0.898	0.899	**0.911**
dam	0.740	0.709	0.739	**0.755**	0.732
golffield	0.816	0.811	**0.823**	0.810	0.817
groundtrackfield	0.802	0.806	0.805	0.802	**0.813**
harbor	**0.749**	**0.749**	0.740	0.743	0.741
overpass	**0.682**	0.670	0.666	0.677	**0.682**
ship	0.940	0.943	0.939	0.941	**0.945**
stadium	**0.946**	0.940	0.937	0.931	0.933
storagetank	0.842	**0.845**	0.844	0.840	0.841
tenniscourt	0.935	0.931	0.935	0.928	**0.941**
trainstation	0.622	0.624	0.614	0.608	**0.642**
vehicle	0.749	**0.758**	0.748	0.755	0.752
windmill	0.814	**0.832**	0.828	0.831	0.821
**mAP50**	0.801	0.801	0.798	0.800	**0.804**

*Note:* The AP@0.5 values in this table are reported from one representative seed and are used only for per-class analysis. Bold values indicate the highest value in each row.

**Table 5:** Three-seed stability comparison of different teacher-weighting strategies on DIOR.

Setting	Seed 1	Seed 2	Seed 3	Mean ± Std	Mean Gain over Static 3T	Seed-Wise Wins over Static 3T
Static 3T	0.8006	0.7996	0.8001	0.8001±0.0004	–	–
Dynamic_3T_KD	0.8034	0.8033	0.8031	0.8031±0.0003	+0.0030	3/3
Semantic_only	0.8021	0.8032	0.8025	0.8026±0.0004	–	–
Topo_only	0.8018	0.8016	0.8025	0.8020±0.0003	–	–
LGR_only	0.7985	0.7996	0.7999	0.7993±0.0006	–	–

*Note:* All results are mAP50 on the DIOR validation set. Dynamic_3T_KD and Static 3T use the same teacher combination and training protocol; the only difference is whether the teacher weights are dynamically estimated or statically fixed.

**Table 6:** Category-wise teacher preference statistics for the classification branch on DIOR.

Class	Semantic	LGR	Topo	Dominant Teacher
Expressway-Service-area	0.356	0.323	0.321	Semantic
Expressway-toll-station	0.459	0.273	0.268	Semantic
airplane	0.432	0.282	0.286	Semantic
airport	0.374	0.321	0.305	Semantic
baseballfield	0.429	0.293	0.278	Semantic
basketballcourt	0.438	0.276	0.286	Semantic
bridge	0.373	0.318	0.310	Semantic
chimney	0.336	0.315	0.349	Topo
dam	0.370	0.319	0.311	Semantic
golffield	0.351	0.330	0.319	Semantic
groundtrackfield	0.416	0.300	0.284	Semantic
harbor	0.364	0.321	0.315	Semantic
overpass	0.371	0.318	0.311	Semantic
ship	0.362	0.321	0.317	Semantic
stadium	0.363	0.330	0.307	Semantic
storagetank	0.156	0.443	0.401	LGR
tenniscourt	0.594	0.203	0.203	Semantic
trainstation	0.365	0.314	0.321	Semantic
vehicle	0.413	0.294	0.293	Semantic
windmill	0.465	0.268	0.268	Semantic

**Table 7:** Category-wise teacher preference statistics for the regression branch on DIOR.

Class	Semantic	LGR	Topo	Dominant Teacher
Expressway-Service-area	0.298	0.286	0.416	Topo
Expressway-toll-station	0.491	0.215	0.294	Semantic
airplane	0.310	0.259	0.431	Topo
airport	0.285	0.402	0.312	LGR
baseballfield	0.476	0.202	0.322	Semantic
basketballcourt	0.284	0.312	0.404	Topo
bridge	0.227	0.365	0.408	Topo
chimney	0.282	0.295	0.423	Topo
dam	0.280	0.400	0.320	LGR
golffield	0.281	0.369	0.349	LGR
groundtrackfield	0.268	0.433	0.299	LGR
harbor	0.278	0.348	0.374	Topo
overpass	0.217	0.382	0.401	Topo
ship	0.270	0.307	0.423	Topo
stadium	0.415	0.278	0.307	Semantic
storagetank	0.173	0.418	0.410	LGR
tenniscourt	0.358	0.262	0.380	Topo
trainstation	0.296	0.293	0.412	Topo
vehicle	0.377	0.243	0.381	Topo
windmill	0.337	0.304	0.359	Topo

**Table 8:** Definition of attribute-oriented category groups and compared distillation settings on DIOR.

Item	Definition	Selection Criterion/Distillation Setting	Included Categories or Meaning
Small-target group	Categories with relatively small object scale.	Top 5 categories with the smallest median normalized object area $A_{c}$.	vehicle, ship, windmill, bridge, Expressway-toll-station
Elongated-target group	Categories with obvious elongated shape characteristics.	Top 5 categories with the largest median elongation ratio $R_{c}$.	train station, ship, harbor, tennis court, vehicle
Complex-background group	Categories located in relatively cluttered surrounding regions.	Top 5 categories with the largest median outer-ring entropy $E_{c}$.	stadium, train station, Expressway-toll-station, storage tank, chimney
Sem. T	Semantic-teacher-only distillation.	The student is distilled only from the semantic classification teacher.	Used to evaluate the contribution of semantic classification knowledge.
LGR. T	Geometric-teacher-only distillation.	The student is distilled only from the local geometric regression teacher.	Used to evaluate the contribution of geometric localization knowledge.
Topo. T	Topology-teacher-only distillation.	The student is distilled only from the structural topology teacher.	Used to evaluate the contribution of contextual topology knowledge.
Static 3T	Static three-teacher distillation.	The student receives supervision from the three teachers with fixed weights.	Used to evaluate naive multi-teacher fusion without adaptive weighting.
Dynamic 3T	Dynamic three-teacher distillation.	The student adaptively integrates semantic, geometric, and topology teacher knowledge.	The proposed dynamic heterogeneous multi-teacher distillation.

*Note:* Sem. T, LGR. T, and Topo. T denote the student models distilled only by the semantic classification teacher, local geometric regression teacher, and structural topology teacher, respectively. Static 3T denotes fixed-weight fusion of the three teachers, while Dynamic 3T denotes the proposed adaptive three-teacher distillation. The three attribute groups are not mutually exclusive because they are constructed according to independent category attributes.

**Table 9:** Group-wise mAP50 comparison and absolute gains on DIOR.

Attribute Group	Sem. T	LGR. T	Topo. T	Baseline	Static 3T	Dynamic 3T
Small-target group	0.7213	0.7312	0.7495	0.7353	0.7345	0.7434
Elongated-target group	0.8042	0.8121	0.8027	0.8008	0.7973	0.8106
Complex-background group	0.8086	0.7789	0.8056	0.7882	0.7879	0.8049
Mean	0.7780	0.7741	0.7859	0.7748	0.7732	0.7863

**Table 10:** Comparison with existing lightweight detectors on DIOR.

Method	Params (M)	GFLOPs	mAP50
YOLO11n_baseline (Ours)	2.594	6.461	0.8013
**Dynamic_3T_KD (Ours)**	**2.418**	**5.563**	**0.8036**
YOLOv5s	7.02	15.8	0.804
Improved YOLOv5s	9.67	18.4	0.816
YOLOv8n	3.0	8.1	0.747
YOLOv8n-CTAM	4.7	11.2	0.765
YOLOv5s (reproduced)	9.1	24.0	0.817
YOLOv8n (reproduced)	3.0	8.1	0.789
YOLOv26n (reproduced)	2.512	5.81	0.802

*Note:* Results of YOLOv5s, Improved YOLOv5s, YOLOv8n, and YOLOv8n-CTAM without the reproduced mark are taken from the original papers or public sources. Bold values indicate the results of the final model.

**Table 11:** Group-wise AP50 comparison with representative knowledge distillation baselines on DIOR.

Attribute Group	Baseline	Static 3T	Dynamic 3T	Feature KD	Masked KD
Small-target group	0.7353	0.7345	0.7434	0.7468	**0.7513**
Elongated-target group	0.8008	0.7973	**0.8106**	0.8013	0.8033
Complex-background group	0.7882	0.7879	**0.8049**	0.7886	0.7981
Mean	0.7748	0.7732	**0.7863**	0.7789	0.7842

*Note:* Feature KD and Masked KD are implemented as representative feature-based and mask-based single-teacher distillation baselines, respectively. Both baselines use the topology teacher as the teacher model, since the topology-teacher-only setting obtains the best mAP50 among the three single-teacher variants. Static 3T denotes fixed-weight fusion of the semantic, geometric, and topology teachers, while Dynamic 3T denotes the proposed adaptive heterogeneous multi-teacher distillation. The best result in each row is highlighted in bold. Bold values indicate the highest value in each row.

**Table 12:** Cross-dataset generalization results on NWPU VHR-10.

Dataset	Method	Seed 1	Seed 2	Seed 3	Mean	Std	Gain vs. Baseline
VHR-10	YOLO11n_baseline	0.91445	0.90603	0.90101	0.90716	0.00555	–
VHR-10	Dynamic_3T_KD	0.89631	0.92876	0.92000	0.91502	0.01371	+0.00786
VHR-10	LGRDetectP4	0.89829	0.92068	0.90753	0.90883	0.00918	+0.00167
VHR-10	Static 3T	0.90628	0.88749	0.90517	0.89964	0.00860	−0.00742

**Table 13:** Deployment efficiency comparison under PT, ONNX, and TensorRT FP32 static formats.

Model	Latency (ms)	FPS	Model Size (MB)	Avg. GPU Utilization (%)	Peak GPU Memory Usage (MiB)
Baseline-PT	22.945	43.58	5.23	36.80	438.6
**Ours-PT**	16.332	61.23	4.96	36.64	438.6
Baseline-ONNX	34.977	28.59	10.13	36.02	442.6
**Ours-ONNX**	15.517	64.45	9.50	37.74	442.6
Baseline-TensorRT FP32 static	22.998	43.48	12.96	35.27	414.6
**Ours-TensorRT FP32 static**	12.950	77.22	11.58	36.59	414.6

*Note:* All latency and FPS results are measured with batch size 1 and an input size of 640×640 on an RTX 4060 Laptop GPU. Avg. GPU utilization denotes the average GPU utilization during inference, while peak GPU memory usage denotes the maximum observed device memory usage, including runtime and inference-framework overhead.

## Data Availability

The datasets used in this study are publicly available and have been widely used for optical remote sensing object detection evaluation. The DIOR dataset can be obtained from its official website: https://opendatalab.org.cn/OpenDataLab/DIOR (accessed on 19 June 2026). The NWPU VHR-10 dataset can be obtained from its official website: https://opendatalab.org.cn/OpenDataLab/NWPU_VHR-10 (accessed on 19 June 2026).

## Abbreviations

The following abbreviations are used in this manuscript:

DIOR	Dataset for Object Detection in Optical Remote Sensing Images
DFL	Distribution Focal Loss
FPS	Frames Per Second
GFLOPs	Giga Floating-Point Operations
IoU	Intersection over Union
KD	Knowledge Distillation
LGR	Lightweight Geometric Regression
mAP	Mean Average Precision
ONNX	Open Neural Network Exchange
PT	PyTorch
TensorRT	TensorRT Inference Runtime
